# A Fully Implicit Log-Conformation Tensor Coupled Algorithm for the Solution of Incompressible Non-Isothermal Viscoelastic Flows

**DOI:** 10.3390/polym14194099

**Published:** 2022-09-30

**Authors:** Célio Fernandes

**Affiliations:** LASI—Associate Laboratory of Intelligent Systems, IPC—Institute for Polymers and Composites, Polymer Engineering Department, School of Engineering, Campus de Azurém, University of Minho, 4800-058 Guimarães, Portugal; cbpf@dep.uminho.pt

**Keywords:** fully implicit coupled solver, viscoelastic flow, log-conformation tensor approach, non-isothermal effects, finite volume method, OpenFOAM

## Abstract

In this work, a block-coupled algorithm is presented, which can compute laminar, incompressible, non-isothermal, viscoelastic flow problems based on the log-conformation tensor approach. The inter-equation coupling of the incompressible Cauchy linear momentum and mass conservation equations is obtained in a procedure based on the Rhie–Chow interpolation. The divergence of the log-conformation tensor term in the linear momentum equations is implicitly discretized in this work. In addition, the velocity field is considered implicitly in the log-conformation tensor constitutive equations by expanding the advection, rotation and the rate of deformation terms with a Taylor series expansion truncated at the second-order error term. Finally, the advection and diffusion terms in the energy equation are also implicitly discretized. The mass, linear momentum, log-conformation tensor constitutive model and energy-discretized linear equations are joined into a block-matrix following a monolithic framework. Validation of the newly developed algorithm is performed for the non-isothermal viscoelastic matrix-based Oldroyd-B fluid flow in the axisymmetric 4:1 planar sudden contraction benchmark problem.

## 1. Introduction

The polymers’ processing techniques are predominantly non-isothermal, such as injection molding [1,2,3], heat exchange problems [4,5], or in plastication, including heating and cooling sequences [6,7]. The thermal conductivity and heat transfer are usually low in this processes; however, due to the heating or cooling of the machine’s operations, large temperature gradients arise in the fluid [4,8]. In addition, the viscoelastic behavior of polymers acts on the temperature field as well as on the fluid deformation [4,8]. Therefore, flow properties are strongly dependent on both rheology and temperature; and, thus, it is essential to understand and make predictions regarding non-isothermal viscoelastic fluid flows.

The temperature dependence of linear viscoelastic properties (such as the relaxation time λ) can be included in constitutive equations using the time-temperature superposition principle [9]. In this way, the material properties can be defined through a function of the temperature, the so-called shift factor [10]. Two empirical correlations of the shift factor are widely employed: the William–Landel-Ferry (WLF) [11] and Arrhenius [12] models. Thus, the temperature is considered an independent variable in the constitutive equations employed to compute the components of the polymeric stress tensor (see the work of Peters and Baaijens [13] for a detailed discussion on this topic). In addition, when solving non-isothermal viscoelastic flows, the internal energy of the fluid is not only a function of the temperature [13]. The conversion mechanisms of internal energy need to be taken into account for non-isothermal viscoelastic flows; specifically, the thermal energy is partly dissipated and partly stored in the fluid. Therefore, the energy equation should predict which part of the mechanical power is dissipated and which part is accumulated as elastic energy [4,8]. For that purpose, an additional term is needed in the energy equation [14]. Peters and Baaijens [13] developed an internal energy equation for multiple rate-type fluids based on a constant weighting factor that characterizes the ratio of entropy to energy elasticity [15]. Several numerical studies have also used this concept [16,17,18] and we will employ this in the current work.

Numerical simulations can describe these complex flow mechanisms and help to gain a better understanding of, and improvements in, the processes where they occur. For that purpose, Computational Fluid Dynamics (CFD) are used to guide the theoretical researchers and practitioner engineers, through the use of both open-source [3,4,5,7,19] and proprietary software [20]. In the last decade, a significant effort has been made in research on the non-isothermal flows of viscoelastic fluids. A survey of the scientific literature finds different works that describe the non-isothermal viscoelastic fluid flows based on iterative numerical algorithms. For example, Shahbani-Zahiri et al. [21] studied the recirculation and thermal regions of viscoelastic flow in the symmetric planar problem for different expansion angles. Kunisch and Marduel [22] employed the finite element (FE) method to study the optimal control of non-isothermal viscoelastic fluids to minimize vortices and control the heat flux. Spanjaards et al. [23] performed a 3D transient non-isothermal simulation to predict the extrudate shape of viscoelastic fluids emerging from an asymmetric keyhole-shaped die. However, the current state-of-the-art codes depend on iterative algorithms, such as the Semi-Implicit Method for Pressure Linked Equations (SIMPLE) procedure [24], which are known to delay the convergence of the problem of interest when compared to monolithic or coupled algorithms [25,26,27,28,29,30]. The iterative algorithms, also known as segregated algorithms, are characterized to provide a separate solution of the linear momentum, mass, viscoelastic polymer stress tensor and energy conservation equations, which are then iterated until convergence. Recently, with the increase in computational resources and due to scalability problems in the segregated algorithms, the monolithic approach has been used, with the advantage of decreasing the computational wall time of the simulation, particularly for finer meshes, as shown by Fernandes et al. [28]. Thus, a methodology based on the monolithic approach for the simulation of non-isothermal viscoelastic flows would be of major importance.

In addition, the benchmark problems of both planar and axisymmetric contraction flows are also extensively studied to evaluate the stability of newly developed numerical algorithms [31,32,33]. These benchmark problems are especially important because, near the contraction, complex flow profiles are generated, and thus large stress gradients are developed, which can cause numerical difficulties, leading to the overall failure of the algorithms. Bearing this in mind, we will revisit the axisymmetric sudden contraction benchmark flow to validate the newly developed, fully implicit, coupled solver for non-isothermal viscoelastic fluid flows.

The rheological data available in the literature for the validation of non-isothermal viscoelastic fluids are scarse due to the complex fluid behaviour, which generally requires several modes to capture the full range of operating conditions. In this work, we will employ the highly elastic, polyisobutylene-based polymer solution (PIB-Boger fluid), which is typically described by the quasi-linear Oldroyd-B viscoelastic fluid model. Another important issue to consider when solving viscoelastic fluid flows is the elastic effects, specifically, the flow at high Weissenberg numbers [34]. It is well known that numerical simulations tend to become unstable at increased Weissenberg numbers, the so-called High Weissenberg Number Problem (HWNP). The seminal work of Fattal and Kupferman [35] proposed a reformulation of the viscoelastic stress-tensor-based formulation to solve the HWNP, where the logarithm of the conformation tensor is used as the main variable in the constitutive transport equation. Different methods have been also used to solve the HWNP, such as the square root of the conformation tensor [36]. A detailed discussion of this topic can be found in Afonso et al. [37]. In this work, we will employ the log-conformation tensor approach to handle the HWNP.

In this manuscript, a new numerical code is developed in the context of the Finite Volume Method (FVM), following a monolithic framework to compute the non-isothermal flow of viscoelastic fluids. To the author’s knowledge, the other CFD codes, which provide a fully implicit block-coupled solution for a discretized, log-conformation, viscoelastic, fluid-flow system, were developed by Knechtges [38] using the Finite Element Method (FEM) and Spahn [39] using FVM; however, these studies did not consider the non-isothermal effects. In this work, the solution to the enlarged system of equations, composed of continuity, linear momentum, log-conformation tensor constitutive equation and energy, is obtained using a Bi-Conjugate Gradient Stabilized solver. The validation of the fully implicit, block-coupled, non-isothermal, viscoelastic, log-conformation tensor-based solver is performed for the Oldroyd-B fluid flow in the axisymmetric 4:1 planar sudden-contraction benchmark problem. For assessment purposes, the results obtained with the newly-developed code are compared with numerical results found in the scientific literature. We study flows at a high Weissenberg number and we investigate the limits of pure energy elasticity and pure entropy elasticity. Lastly, we also analyzed the effect of the jump in wall temperature near the contraction for positive and negative increments.

The remaining sections of the manuscript are organized as follows. In Section 2, the governing equations for the stress tensor and log-conformation tensor-based formulations of non-isothermal viscoelastic flows are presented. Subsequently, in Section 3, the numerical procedure of the block-coupled algorithm will be described in detail, including the finite-volume discretization process for all the implicit terms considered in the governing equations. In Section 4, the validation of the newly-developed numerical algorithm is performed, and in Section 5 the main conclusions of the work are addressed.

## 2. Governing Equations

In this section, the equations that involve non-isothermal viscoelastic fluid flow problems are presented for both stress-tensor- and log-conformation tensor-based formulations.

### 2.1. Stress-Tensor-Based Formulation

The governing equations for laminar, incompressible, non-isothermal viscoelastic flows are the conservation of mass and linear momentum, together with a constitutive equation modeling the polymer stress behavior and the energy equation to account for thermodynamical effects.

The conservation of mass and linear momentum equations read as follows:

(1) $$\begin{array}{c} \frac{\partial u_{i}}{\partial x_{i}}=0, \end{array}$$

(2) $$\begin{array}{c} \rho \left(\frac{\partial u_{i}}{\partial t}+u_{j}\frac{\partial u_{i}}{\partial x_{j}}\right)+\frac{\partial p}{\partial x_{i}}-\frac{\partial \tau_{ij}}{\partial x_{j}}=0, \end{array}$$

where Einstein’s summation convention applies, $u_{i}$ are the velocity components along the Cartesian co-ordinates $x_{i}$, ρ is the fluid density, *t* is the time, *p* is the pressure and $\tau_{ij}$ are the components of the total extra-stress tensor (i,j=1,2 for 2D flows), which is split into Newtonian (solvent), ${\left(\tau_{N}\right)}_{ij}$, and elastic (polymeric), ${\left(\tau_{E}\right)}_{ij}$, contributions, such that $\tau_{ij}={\left(\tau_{N}\right)}_{ij}+{\left(\tau_{E}\right)}_{ij}$.

The calculation of the stress terms is completed using the following relations:

(3) $$\begin{array}{c} {\left(\tau_{N}\right)}_{ij}=2\eta_{N}\left(T\right)D_{ij}=\eta_{N}\left(T\right)\left(\frac{\partial u_{i}}{\partial x_{j}}+\frac{\partial u_{j}}{\partial x_{i}}\right), \end{array}$$

(4) $$\begin{array}{c} \begin{array}{cc} & \frac{1}{\eta_{E}\left(T\right)}\left(I_{ij}+h\left({\left(\tau_{E}\right)}_{ij}\right)\right){\left(\tau_{E}\right)}_{ij}+\frac{\lambda (T)}{\eta_{E}\left(T\right)}\left(\frac{\partial {\left(\tau_{E}\right)}_{ij}}{\partial t}+u_{k}\frac{\partial {\left(\tau_{E}\right)}_{ij}}{\partial x_{k}}\right)-\left(\frac{\partial u_{i}}{\partial x_{j}}+\frac{\partial u_{j}}{\partial x_{i}}\right)-\\ & \frac{\lambda (T)}{\eta_{E}\left(T\right)}\left({\left(\tau_{E}\right)}_{ik}\frac{\partial u_{j}}{\partial x_{k}}+{\left(\tau_{E}\right)}_{jk}\frac{\partial u_{i}}{\partial x_{k}}\right)=0, \end{array} \end{array}$$

where $\eta_{N}\left(T\right)$ and $\eta_{E}\left(T\right)$ are the temperature-dependent solvent and polymeric viscosities, respectively, $D_{ij}$ is the strain rate tensor, which describes the rate of stretching and shearing, λ(T) is the temperature-dependent polymer relaxation time, $I_{ij}$ is the identity tensor and $h({\left(\tau_{E}\right)}_{ij})$ is a tensor that can be given by different expressions, related to the constitutive equation chosen to model the viscoelastic fluid. For the Oldroyd-B model [40], $h({\left(\tau_{E}\right)}_{ij})=0$. For the Giesekus model [41] $h\left({\left(\tau_{E}\right)}_{ij}\right)=\frac{\kappa \lambda }{\eta_{E}}{\left(\tau_{E}\right)}_{ij}$, where κ is a positive constant, the so-called mobility factor, which is related to the elongational behavior of the fluids. For the Phan–Thien–Tanner (PTT) model [42,43], the tensor h is of the form $h\left({\left(\tau_{E}\right)}_{ij}\right)=\frac{\epsilon \lambda }{\eta_{E}}\mathrm{tr}\left({\left(\tau_{E}\right)}_{ij}\right)I_{ij}$, where $\mathrm{tr}\left({\left(\tau_{E}\right)}_{ij}\right)={\left(\tau_{E}\right)}_{ii}$ is the trace of the polymeric stress tensor and ϵ is a material parameter called the extensibility factor, related to the fluid behavior in extensional flow. In addition, the Giesekus and PTT models present one more non-linearity, which is given by the product $h\left({\left(\tau_{E}\right)}_{ij}\right){\left(\tau_{E}\right)}_{ij}$. This term is responsible for the shear-thinning, the non-zero second normal stress coefficient and the stress overshoot in transient shear flows of viscoelastic fluids. In this work, we will provide a preliminary assessment of the merits of the fully implicit, block-coupled, non-isothermal, viscoelastic, log-conformation tensor-based algorithm for calculations using the Oldroyd-B fluid model, which is commonly used to validate newly-developed viscoelastic codes due to the stress singular behavior near sharp corners or at stagnation points. For these models, a characteristic (solvent) viscosity ratio can be defined by $\beta =\eta_{N}/\left(\eta_{N}+\eta_{E}\right)=\eta_{N}/\eta_{0}$, known as the retardation ratio, where $\eta_{0}$ is the total viscosity in the limit of vanishing shear rate.

Following the work of Peters and Baaijens [13] the energy balance equation for the case of viscoelastic flows is as follows:

(5) $$\begin{array}{c} \rho C_{p}\left(\frac{\partial T}{\partial t}+u_{i}\frac{\partial T}{\partial x_{i}}\right)-k\frac{\partial^{2}T}{\partial x_{i}^{2}}={\left(\tau_{N}\right)}_{ij}D_{ji}+\alpha {\left(\tau_{E}\right)}_{ij}D_{ji}+\left(1-\alpha \right)\frac{{\left(\tau_{E}\right)}_{ii}}{2\bar{\lambda }\left(T\right)}, \end{array}$$

where *k* is the thermal conductivity of the fluid, without dependence on temperature *T* and polymer orientation, $C_{p}$ is the specific heat capacity, also without temperature and polymer orientation dependence [44], and α is the energy partitioning coefficient. When α=1, all mechanical energy is dissipated as heat (pure entropy elasticity), and if α=0, all mechanical energy is stored as elastic energy (pure elastic material) [13,18]. Habla et al. [18] concluded that the effect of the parameter α is negligible because, with a fully developed shear flow, only stress work occurs, and the internal energy storage is absent. In addition, $\bar{\lambda }\left(T\right)=\lambda \left(T\right){\left(1+\frac{\lambda \left(T\right)\;\epsilon \;{\left(\tau_{E}\right)}_{ii}}{\eta_{E}\left(T\right)}\right)}^{-1}$ for the PTT model and $\bar{\lambda }\left(T\right)=\lambda \left(T\right){\left(1+\frac{\lambda \left(T\right)\;\kappa \;{\left(\tau_{E}\right)}_{ij}}{\eta_{E}\left(T\right)}\right)}^{-1}$ for the Giesekus model. For the Oldroyd-B model calculations considered in the validation section of this work (Section 4), $\bar{\lambda }\left(T\right)=\lambda \left(T\right)$. The temperature dependencies of the relaxation time, λ(T), solvent and polymeric viscosities, $\eta_{N}\left(T\right)$ and $\eta_{E}\left(T\right)$, respectively, are given by

(6) $$\begin{array}{c} \lambda \left(T\right)=a_{T}\lambda \left(T_{0}\right), \end{array}$$

(7) $$\begin{array}{c} \eta_{N}\left(T\right)=a_{T}\eta_{N}\left(T_{0}\right), \end{array}$$

(8) $$\begin{array}{c} \eta_{E}\left(T\right)=a_{T}\eta_{E}\left(T_{0}\right), \end{array}$$

where $T_{0}$ is a reference temperature and $a_{T}$ is a shift factor obeying the Williams–Landel–Ferry (WLF) relation:

(9) $$\begin{array}{c} a_{T}=\exp\left(\frac{-C_{1}\left(T-T_{0}\right)}{C_{2}+T-T_{0}}\right), \end{array}$$

in which $C_{1}$ and $C_{2}$ are the WLF parameters and $T_{0}$ is the reference temperature. Frequently used sets of WLF parameters $\left(C_{1},C_{2}\right)$ are (5, 150) for temperatures relatively far from the glass transition temperature $T_{g}$, enabling the thermorheological coupling, and (15, 50) for temperatures near $T_{g}$ [18].

### 2.2. Log-Conformation Tensor-Based Formulation

In this section, we write the viscoelastic stress tensor-based formulation in terms of the log-conformation tensor variable, which was proposed by Fattal and Kupferman [35] to address the HWNP. For that purpose, the polymeric stress tensor, ${\left(\tau_{E}\right)}_{ij}$, is related to the conformation tensor, $\sigma_{ij}$, by the following equation

(10) $$\begin{array}{c} {\left(\tau_{E}\right)}_{ij}=\frac{\eta_{E}\left(T\right)}{\lambda (T)}\left(\sigma_{ij}-I_{ij}\right). \end{array}$$

Subsequently, the conformation tensor $\sigma_{ij}$ is replaced by its matrix logarithmic $\Psi_{ij}=\log\left(\sigma_{ij}\right)$, and Equations (2), (4) and (5) are substituted by

(11) $$\begin{array}{c} \rho \left(\frac{\partial u_{i}}{\partial t}+u_{j}\frac{\partial u_{i}}{\partial x_{j}}\right)+\frac{\partial p}{\partial x_{i}}-2\eta_{N}\left(T\right)\frac{\partial D_{ij}}{\partial x_{j}}-\frac{\eta_{E}\left(T\right)}{\lambda (T)}\frac{\partial \left(e^{\Psi_{ij}}-I_{ij}\right)}{\partial x_{j}}=0, \end{array}$$

(12) $$\begin{array}{c} \frac{1}{\lambda (T)}\left[\left(I_{ij}+h\left(e^{\Psi_{ij}}\right)\right)\left(e^{\Psi_{ij}}-I_{ij}\right)\right]+\frac{\partial e^{\Psi_{ij}}}{\partial t}+u_{k}\frac{\partial e^{\Psi_{ij}}}{\partial x_{k}}-e^{\Psi_{ik}}\frac{\partial u_{j}}{\partial x_{k}}-e^{\Psi_{jk}}\frac{\partial u_{i}}{\partial x_{k}}=0, \end{array}$$

(13) $$\begin{array}{c} \rho C_{p}\left(\frac{\partial T}{\partial t}+u_{i}\frac{\partial T}{\partial x_{i}}\right)-k\frac{\partial^{2}T}{\partial x_{i}^{2}}={\left(\tau_{N}\right)}_{ij}D_{ji}+\frac{\eta_{E}\left(T\right)}{\lambda (T)}\left[\alpha \left(e^{\Psi_{ij}}-I_{ij}\right)D_{ji}+\left(1-\alpha \right)\frac{{\left(e^{\Psi }-I\right)}_{ii}}{2\bar{\lambda }\left(T\right)}\right], \end{array}$$

with $\bar{\lambda }\left(T\right)=\lambda \left(T\right){\left(1+\epsilon \;{\left(e^{\Psi }-I\right)}_{ii}\right)}^{-1}$ for the PTT model, $\bar{\lambda }\left(T\right)=\lambda \left(T\right){\left(1+\kappa \;{\left(e^{\Psi }-I\right)}_{ij}\right)}^{-1}$ for the Giesekus model and $\bar{\lambda }\left(T\right)=\lambda \left(T\right)$ for the Oldroyd-B model. Here, $e^{\Psi_{ij}}$ is the matrix exponential function, defined as $e^{\Psi_{ij}}=\sum_{m=1}^{d}e^{\xi_{m}}P_{m}$, with *d* as the dimension of the physical space (d=2 for the calculations performed in this work), $\xi_{m}$ the eigenvalues of $\Psi_{ij}$ and $P_{m}$ is the projection matrix onto the corresponding eigenspace. Therefore, if ${\hat{e}}_{m}$ is the eigenvector corresponding to $\xi_{m}$, then $P_{m}={\hat{e}}_{m}⊗{\hat{e}}_{m}$ [38]. In addition, note that $\lambda (T_{0})$ and $\eta_{P}\left(T_{0}\right)$ are known values for the reference temperature $T_{0}$, and, therefore, the quotient $\frac{\eta_{E}\left(T\right)}{\lambda (T)}$ in Equations (11) and (13) is a constant, considering that λ and $\eta_{E}$ scale in the same way [45].

## 3. Numerical Method

In this section, we will describe a finite volume numerical method to set up a block-coupled solver procedure to simultaneously solve the continuity (Equation (1)), linear momentum (Equation (11)), log-conformation tensor (Equation (12)) and energy (Equation (13)) equations.

Within the framework of the block-coupled solver developed in this work, the advection, pressure gradient, diffusion and log-conformation tensor terms in the conservation of linear momentum equations are implicitly discretized (see Section 3.1). Subsequently, the velocity field term in the conservation of mass equation is also treated in an implicit manner (see Section 3.2). In addition, and as an extension to our previous work [28], where we have discretized implicitly the advection term in the stress constitutive equation, here the rotation and the rate of deformation terms are implicitly discretized (see Section 3.3). Lastly, the advection and diffusion terms in the energy equation are also implicitly discretized (see Section 3.4). The rate of change term in all the equations is implicitly discretized using the backward implicit Euler scheme.

### 3.1. Discretization of the Equations for Conservation of Linear Momentum

**Figure 1:**
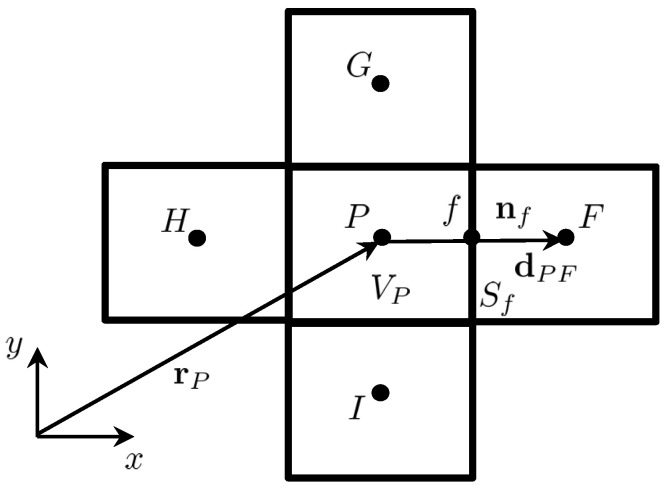
Schematic representation of the control volume $V_{P}$ with centroid *P* (owner), with distance vector to the origin $r_{P}$, and neighboring control volumes with centroids F,G,H and *I*. The face shared by the control volumes with centroids *P* and *F* is represented by *f*, with area $S_{f}$ and face unit normal vector $n_{f}$. The distance vector between *P* and *F* is represented by $d_{PF}$.

In the framework of the FVM, the discretization process starts by integrating the conservation of linear momentum equations (Equation (11)) over a general control volume (also called *representative volume* or *cell*) $V_{P}$, where the subscript *P* refers to values of the variables at cell with centroid *P*, as shown in Figure 1, to yield

(14) $$\begin{array}{c} \begin{array}{cc} & \rho \left(\int_{V_{P}}\frac{\partial u_{i}}{\partial t}dV_{P}+\int_{V_{P}}u_{j}\frac{\partial u_{i}}{\partial x_{j}}dV_{P}\right)+\int_{V_{P}}\frac{\partial p}{\partial x_{i}}dV_{P}-\int_{V_{P}}\left(\eta_{N}\left(T\right)+\eta^{★}\right)\frac{\partial^{2}u_{i}}{\partial x_{j}^{2}}dV_{P}-\\ & -\int_{V_{P}}\frac{\eta_{E}\left(T\right)}{\lambda (T)}\frac{\partial {\left(e^{\Psi }-I\right)}_{ij}}{\partial x_{j}}dV_{P}=-\int_{V_{P}}\frac{\partial }{\partial x_{j}}\left(\eta^{★}\frac{\partial u_{i}}{\partial x_{j}}\right)dV_{P}, \end{array} \end{array}$$

where the additional terms involving $\eta^{★}$ are related to the improved both-side diffusion technique [46], which can solve the checkerboard pattern due to numerical instabilities caused by a velocity–stress decoupling. Note that we also used the identity $\frac{\partial }{\partial x_{j}}\left(\frac{\partial u_{j}}{\partial x_{i}}\right)=0$.

The following step of the discretization process is to apply the Gauss divergence theorem to transform the volume integrals of the advection, pressure and diffusion terms in Equation (14) into surface integrals as follows:

(15) $$\begin{array}{c} \begin{array}{cc} & \rho \left(\int_{V_{P}}\frac{\partial u_{i}}{\partial t}dV_{P}+\oint_{S}n_{j}\left(u_{j}u_{i}\right)dS\right)+\oint_{S}n_{i}pdS-\oint_{S}n_{j}\left[\left(\eta_{N}\left(T\right)+\eta^{★}\right)\frac{\partial u_{i}}{\partial x_{j}}\right]dS-\\ & -\int_{V_{P}}\frac{\eta_{E}\left(T\right)}{\lambda (T)}\frac{\partial {\left(e^{\Psi }-I\right)}_{ij}}{\partial x_{j}}dV_{P}=-\oint_{S}n_{j}\eta^{★}\frac{\partial u_{i}}{\partial x_{j}}dS, \end{array} \end{array}$$

where *S* is the boundary of control volume $V_{P}$ and $n_{i}$ are the components of the outward pointing unit vector normal to *S*.

Subsequently, a second-order integration scheme is employed to approximate the surface integrals and the following linear momentum semi-discretized equations are obtained:

(16) $$\begin{array}{c} \begin{array}{cc} & V_{P}\rho_{P}\frac{{\left(u_{i}\right)}_{P}-{\left(u_{i}\right)}_{P}^{0}}{\Delta t}+\sum_{f=nb(P)}{\left(s_{j}\rho u_{j}u_{i}\right)}_{f}+\sum_{f=nb(P)}{\left(s_{i}p\right)}_{f}-\\ & -\sum_{f=nb(P)}{\left[s_{j}\left(\eta_{N}\left(T\right)+\eta^{★}\right)\frac{\partial u_{i}}{\partial x_{j}}\right]}_{f}-\int_{V_{P}}\frac{\eta_{E}\left(T\right)}{\lambda (T)}\frac{\partial {\left(e^{\Psi }-I\right)}_{ij}}{\partial x_{j}}dV_{P}\\ = & -\sum_{f=nb(P)}{\left(s_{j}\eta^{★}\frac{\partial u_{i}}{\partial x_{j}}\right)}_{f}, \end{array} \end{array}$$

where Δt is the time-step, the superscript 0 represents the previous time step value, nb refers to values at the faces *f*, obtained by interpolation between *P* and its neighbors, and $s_{i}$ are the components of the area normal vector to face *f*.

Finally, the linear momentum semi-discretized equations are transformed into an algebraic linear system of equations by expressing the variation in the dependent variable and its derivatives in terms of the control volume *P* and its neighbors’ values at the respective centroids, such as

(17) $$\begin{array}{c} \left\{\begin{array}{cc} \begin{array}{cc} & a_{P}^{uu}u_{P}+a_{P}^{uv}v_{P}+a_{P}^{up}p_{P}+a_{P}^{u\Psi_{xx}}{\left(\Psi_{xx}\right)}_{P}+a_{P}^{u\Psi_{xy}}{\left(\Psi_{xy}\right)}_{P}+\\ & +\sum_{F=NB(P)}a_{F}^{uu}u_{F}+\sum_{F=NB(P)}a_{F}^{uv}v_{F}+\sum_{F=NB(P)}a_{F}^{up}p_{F}+\\ & +\sum_{F=NB(P)}a_{F}^{u\Psi_{xx}}{\left(\Psi_{xx}\right)}_{F}+\sum_{F=NB(P)}a_{F}^{u\Psi_{xy}}{\left(\Psi_{xy}\right)}_{F}=b_{P}^{u}, \end{array} & \\ \begin{array}{cc} & a_{P}^{vu}u_{P}+a_{P}^{vv}v_{P}+a_{P}^{vp}p_{P}+a_{P}^{v\Psi_{xy}}{\left(\Psi_{xy}\right)}_{P}+a_{P}^{v\Psi_{yy}}{\left(\Psi_{yy}\right)}_{P}+\\ & +\sum_{F=NB(P)}a_{F}^{vu}u_{F}+\sum_{F=NB(P)}a_{F}^{vv}v_{F}+\sum_{F=NB(P)}a_{F}^{vp}p_{F}+\\ & \sum_{F=NB(P)}a_{F}^{v\Psi_{xy}}{\left(\Psi_{xy}\right)}_{F}+\sum_{F=NB(P)}a_{F}^{v\Psi_{yy}}{\left(\Psi_{yy}\right)}_{F}=b_{P}^{v}, \end{array} \end{array}\right. \end{array}$$

where $a_{P}^{u_{i}\phi }$ and $a_{F}^{u_{i}\phi }$ are the owner and neighbor coefficients in the discretized linear momentum equation representing the velocity component $u_{i}$ and the variable ϕ interactions, respectively; $b_{P}^{u_{i}}$ is the source term, where NB(P) refers to the neighbors of the control-volume with centroid *P*.

For the sake of conciseness, the contributions of the rate of change, advection, pressure gradient, implicit diffusion and explicit diffusion terms shown in Equation (16) can be found in Fernandes et al. [28]. Regarding the contribution of the log-conformation tensor term, $\frac{\partial {\left(e^{\Psi }-I\right)}_{ij}}{\partial x_{j}}$, to the linear momentum equations, we will employ an implicit discretization by considering the following Taylor approximation for the functional ${\left(e^{\Psi }-I\right)}_{ij}$ [28,39]

(18) $$\begin{array}{c} \begin{array}{cc} {\left(e^{\Psi }-I\right)}_{ij} & \approx {\left(e^{\Psi }-I\right)}_{ij}^{0}+\frac{\partial {\left(e^{\Psi }-I\right)}_{ij}}{\partial \Psi_{kl}}{|}_{\Psi_{kl}^{0}}\left(\Psi_{kl}-\Psi_{kl}^{0}\right)\\ & ={\left(e^{\Psi }-I\right)}_{ij}^{0}+\sum_{i,j}^{d}e^{\lambda_{m}/2}e^{\lambda_{n}/2}\frac{\sinh(\left(\lambda_{m}-\lambda_{n}\right)/2)}{(\lambda_{m}-\lambda_{n})/2}\sum_{k,l}p_{ik}^{m}p_{lj}^{n}\left(\Psi_{kl}-\Psi_{kl}^{0}\right), \end{array} \end{array}$$

where the derivative of the functional ${\left(e^{\Psi }-I\right)}_{ij}$ in order to the log-conformation tensor variable is substituted by the expression found in Knechtges [38] for the finite element method, and $p_{ik}^{m}$ and $p_{lj}^{n}$ are the coefficients of the projector matrix belonging to the m-th and n-th eigenvalues $\left(\lambda_{m},\lambda_{n}\right)$ of $\Psi_{ij}^{0}$. Therefore, the divergence of the functional ${\left(e^{\Psi }-I\right)}_{ij}$ can be written as:

(19) $$\begin{array}{c} \begin{array}{cc} & \int_{V_{P}}\left(\frac{\eta_{E}\left(T\right)}{\lambda (T)}\frac{\partial {\left(e^{\Psi }-I\right)}_{ij}}{\partial x_{j}}\right)dV_{P}\\ \approx & \frac{\eta_{E}\left(T\right)}{\lambda (T)}\int_{V_{P}}{\left(\frac{\partial {\left(e^{\Psi }-I\right)}_{ij}}{\partial x_{j}}\right)}^{0}dV_{P}+\frac{\eta_{E}\left(T\right)}{\lambda (T)}\int_{V_{P}}\partial \left[{\left(\frac{\partial {\left(e^{\Psi }-I\right)}_{ij}}{\partial \Psi_{kl}}\right)}^{0}\Psi_{kl}\right]/\partial x_{j}dV_{P}\\ & -\frac{\eta_{E}\left(T\right)}{\lambda (T)}\int_{V_{P}}\partial \left[{\left(\frac{\partial {\left(e^{\Psi }-I\right)}_{ij}}{\partial \Psi_{kl}}\right)}^{0}\Psi_{kl}^{0}\right]/\partial x_{j}dV_{P}, \end{array} \end{array}$$

Subsequently, applying the Gauss divergence theorem we can transform the volume integrals with derivatives into surface integrals

(20a) $$\begin{array}{c} \int_{V_{P}}\partial \left[{\left(\frac{\partial {\left(e^{\Psi }-I\right)}_{ij}}{\partial \Psi_{kl}}\right)}^{0}\Psi_{kl}\right]/\partial x_{j}dV_{P}=\oint_{S}n_{j}{\left(\frac{\partial {\left(e^{\Psi }-I\right)}_{ij}}{\partial \Psi_{kl}}\right)}^{0}\Psi_{kl}dS, \end{array}$$

(20b) $$\begin{array}{c} \int_{V_{P}}\partial \left[{\left(\frac{\partial {\left(e^{\Psi }-I\right)}_{ij}}{\partial \Psi_{kl}}\right)}^{0}\Psi_{kl}^{0}\right]/\partial x_{j}dV_{P}=\oint_{S}n_{j}{\left(\frac{\partial {\left(e^{\Psi }-I\right)}_{ij}}{\partial \Psi_{kl}}\right)}^{0}\Psi_{kl}^{0}dS, \end{array}$$

and obtain the discretized form for the divergence of the functional ${\left(e^{\Psi }-I\right)}_{ij}$ (i.e., the log-conformation tensor term) in the linear momentum equations as follows:

(21) $$\begin{array}{c} \begin{array}{cc} & \int_{V_{P}}\left(\frac{\eta_{E}\left(T\right)}{\lambda (T)}\frac{\partial {\left(e^{\Psi }-I\right)}_{ij}}{\partial x_{j}}\right)dV_{P}\\ \approx & \frac{\eta_{E}\left(T\right)}{\lambda (T)}{\left(\frac{\partial {\left(e^{\Psi }-I\right)}_{ij}}{\partial x_{j}}\right)}^{0}V_{P}+\frac{\eta_{E}\left(T\right)}{\lambda (T)}\sum_{f=nb(P)}{\left(s_{j}{\left(\frac{\partial {\left(e^{\Psi }-I\right)}_{ij}}{\partial \Psi_{kl}}\right)}^{0}\Psi_{kl}\right)}_{f}\\ & -\frac{\eta_{E}\left(T\right)}{\lambda (T)}\sum_{f=nb(P)}{\left(s_{j}{\left(\frac{\partial {\left(e^{\Psi }-I\right)}_{ij}}{\partial \Psi_{kl}}\right)}^{0}\Psi_{kl}^{0}\right)}_{f}. \end{array} \end{array}$$

Lastly, the contributions of the divergence of the log-conformation (DLC) tensor term for the linear momentum equations are given by

(22a) $$\begin{array}{cc} \; & a_{F,DLC}^{u_{j}\psi_{kl}}=-\frac{\eta_{E}\left(T\right)}{\lambda (T)}{\left[s_{j}{\left(\frac{\partial {\left(e^{\Psi }-I\right)}_{ij}}{\partial \Psi_{kl}}\right)}^{0}\right]}_{f}\left(1-w_{f}\right), \end{array}$$

(22b) $$\begin{array}{cc} \; & a_{P,DLC}^{u_{j}\psi_{kl}}=-\frac{\eta_{E}\left(T\right)}{\lambda (T)}\sum_{f=nb(P)}{\left[s_{j}{\left(\frac{\partial {\left(e^{\Psi }-I\right)}_{ij}}{\partial \Psi_{kl}}\right)}^{0}\right]}_{f}w_{f}, \end{array}$$

(22c) $$\begin{array}{cc} \; & b_{P,DLC}^{u_{j}}=-\frac{\eta_{E}\left(T\right)}{\lambda (T)}\left[{\left(\frac{\partial {\left(e^{\Psi }-I\right)}_{ij}}{\partial x_{j}}\right)}^{0}V_{P}-\sum_{f=nb(P)}{\left(s_{j}{\left(\frac{\partial {\left(e^{\Psi }-I\right)}_{ij}}{\partial \Psi_{kl}}\right)}^{0}\Psi_{kl}^{0}\right)}_{f}\right], \end{array}$$

where $w_{f}$ are the interpolation weights.

It should be noted that the total contribution for the owner and neighbor coefficients related to the linear momentum equations’ interactions is given by the sum of the rate of change, advection, pressure gradient, implicit diffusion and divergence of log-conformation tensor terms. In addition, the total contribution for the explicit coefficient related to the linear momentum equations is given by the sum of the rate of change, explicit diffusion and divergence of log-conformation tensor terms.

### 3.2. Discretization of the Equation for Conservation of Mass

Following the same steps as presented in Section 3.1, we begin the discretization of the continuity equation, Equation (1), with the integration over the control volume $V_{P}$ as follows:

(23) $$\begin{array}{c} \int_{V_{P}}\frac{\partial u_{i}}{\partial x_{i}}dV_{P}=0. \end{array}$$

Subsequently, by employing the divergence theorem, the volume integral is transformed into a surface integral as follows:

(24) $$\begin{array}{c} \oint_{S}n_{i}u_{i}dS=0. \end{array}$$

Then, by applying a second-order integration scheme to approximate the surface integral, we can write the semi-discretized form of the continuity equation as:

(25) $$\begin{array}{c} \sum_{f=nb(P)}{\left(s_{i}u_{i}\right)}_{f}=0. \end{array}$$

In a collocated framework, the velocity at the face is obtained by reconstructing a pseudo-momentum equation at the face from the linear momentum equations of the straddling cells *P* and *F*, known as the Rhie–Chow interpolation [47]. For the sake of conciseness, the derivation of the discretized form for the equation of conservation of mass will not be given in detail, but it can be found in our previous work [28], which reads as

(26) $$\begin{array}{c} \sum_{f=nb(P)}{\left[s_{i}\left(-\frac{\partial p}{\partial x_{i}}{\bar{D}}_{i}\right)\right]}_{f}+\sum_{f=nb(P)}{\left(s_{i}{\bar{u}}_{i}\right)}_{f}=\sum_{f=nb(P)}{\left[s_{i}\left(-\bar{\frac{\partial p}{\partial x_{i}}}{\bar{D}}_{i}\right)\right]}_{f}. \end{array}$$

Finally, we can write the mass conservation algebraic equation as:

(27) $$\begin{array}{c} a_{P}^{pp}p_{P}+a_{P}^{pu}u_{P}+a_{P}^{pv}v_{P}+\sum_{F=NB(P)}a_{F}^{pp}p_{F}+\sum_{F=NB(P)}a_{F}^{pu}u_{F}+\sum_{F=NB(P)}a_{F}^{pv}v_{F}=b_{P}^{p}, \end{array}$$

where $a_{P}^{p\phi }$ and $a_{F}^{p\phi }$ are the owner and neighbor coefficients in the discretized mass conservation equation representing the pressure field and the variable ϕ interactions, respectively; $b_{P}^{p}$ is the source term.

The implicit pressure gradient term is discretized (see page 86 of [48]) as follows:

(28) $$\begin{array}{c} {\left[s_{i}\left(-\frac{\partial p}{\partial x_{i}}{\bar{D}}_{i}\right)\right]}_{f}=-\frac{{\left[s_{i}\left(s_{i}{\bar{D}}_{i}\right)\right]}_{f}}{{\left(d_{PF}\right)}_{i}{\left(s_{i}\right)}_{f}}\left(p_{F}-p_{P}\right), \end{array}$$

where the coefficients of the implicit pressure gradient term for the mass conservation equation are given by

(29a) $$\begin{array}{cc} \; & a_{F}^{pp}=-\frac{{\left[s_{i}\left(s_{i}{\bar{D}}_{i}\right)\right]}_{f}}{{\left(d_{PF}\right)}_{i}{\left(s_{i}\right)}_{f}}, \end{array}$$

(29b) $$\begin{array}{cc} \; & a_{P}^{pp}=-\sum_{F=NB(P)}a_{F}^{pp}. \end{array}$$

The implicit coefficients for the second term in Equation (26) (corresponding to the velocity contribution) are given by

(30a) $$\begin{array}{cc} \; & a_{F}^{pu}=s_{f}^{x}\left(1-w_{f}\right),\;a_{F}^{pv}=s_{f}^{y}\left(1-w_{f}\right), \end{array}$$

(30b) $$\begin{array}{cc} \; & a_{P}^{pu}=\sum_{f=nb(P)}s_{f}^{x}w_{f},\;a_{P}^{pv}=\sum_{f=nb(P)}s_{f}^{y}w_{f}. \end{array}$$

Lastly, the coefficients of the explicit pressure gradient term contribution for the mass conservation equation are given by

(31) $$\begin{array}{c} b_{P}^{p}=\sum_{f=nb(P)}{\left[s_{i}\left(-\bar{\frac{\partial p}{\partial x_{i}}}{\bar{D}}_{i}\right)\right]}_{f}. \end{array}$$

### 3.3. Discretization of the Log-Conformation Tensor Constitutive Equations

The constitutive equations, Equation (4), can be written, without loss of generality, for a Giesekus fluid model by (see Theorem A.42 in [38])

(32) $$\begin{array}{c} \begin{array}{cc} & \frac{\partial \Psi_{ij}}{\partial t}+u_{k}\frac{\partial \Psi_{ij}}{\partial x_{k}}+\left[\Psi_{ij},\Omega_{ij}\right]-2f\left(\mathrm{ad}\left(\Psi_{ij}\right)\right)D_{ij}\\ = & -\frac{1}{\lambda (T)}\left(I_{ij}+\kappa \left(e^{\Psi_{ij}}-I_{ij}\right)\right)\left(e^{\Psi_{ij}}-I_{ij}\right)e^{-\Psi_{ij}}, \end{array} \end{array}$$

where $\Omega_{ij}=\left(\frac{\partial u_{i}}{\partial x_{j}}-\frac{\partial u_{j}}{\partial x_{i}}\right)/2$ is the vorticity tensor and $\left[\Psi_{ij},\Omega_{ij}\right]=\Psi_{ij}\Omega_{ij}-\Omega_{ij}\Psi_{ij}$ is the commutator term. Following Knechtges [38], $f(\mathrm{ad}\left(\Psi_{ij}\right))$ is defined by the application of the function $f\left(x\right)=\frac{x/2}{\tanh(x/2)}$ to the adjoint operator $\mathrm{ad}(\Psi_{ij})$, such as

(33) $$\begin{array}{c} f\left(\mathrm{ad}\left(\Psi_{ij}\right)\right)y=\sum_{m,n=1}^{d}f\left(\lambda_{m}-\lambda_{n}\right)P_{m}yP_{n}, \end{array}$$

where *y* is a symmetric matrix satisfying $\mathrm{ad}\left(\Psi_{ij}\right)y=\left[\Psi_{ij},y\right]$, and *y* is continuously differentiable ($C^{1}$). Notice that, Equation (32) simplifies to the constitutive equation for an Oldroyd-B fluid model when κ=0.

The discretization of the log-conformation tensor constitutive equations, Equation (32), starts with the integration over the control volume $V_{P}$ to yield

(34) $$\begin{array}{c} \int_{V_{P}}\frac{\partial \Psi_{ij}}{\partial t}dV_{P}+\int_{V_{P}}u_{k}\frac{\partial \Psi_{ij}}{\partial x_{k}}dV_{P}+\int_{V_{P}}\left[\Psi_{ij},\Omega_{ij}\right]dV_{P}-\int_{V_{P}}2f\left(\mathrm{ad}\left(\Psi_{ij}\right)\right)D_{ij}dV_{P}=\\ -\int_{V_{P}}\frac{1}{\lambda (T)}\left(I_{ij}+\kappa \left(e^{\Psi_{ij}}-I_{ij}\right)\right)\left(e^{\Psi_{ij}}-I_{ij}\right)e^{-\Psi_{ij}}dV_{P}. \end{array}$$

This leads to the algebraic equation with the following form:

(35) $$\begin{array}{c} \left\{\begin{array}{cc} \begin{array}{cc} & a_{P}^{\Psi_{xx}\Psi_{xx}}{\left(\Psi_{xx}\right)}_{P}+a_{P}^{\Psi_{xx}\;u}u_{P}+a_{P}^{\Psi_{xx}\;v}v_{P}+\sum_{F=NB(P)}a_{F}^{\Psi_{xx}\;\Psi_{xx}}{\left(\Psi_{xx}\right)}_{F}+\\ & +\sum_{F=NB(P)}a_{F}^{\Psi_{xx}\;u}u_{F}+\sum_{F=NB(P)}a_{F}^{\Psi_{xx}\;v}v_{F}=b_{P}^{\Psi_{xx}}, \end{array} & \\ \begin{array}{cc} & a_{P}^{\Psi_{xy}\;\Psi_{xy}}{\left(\Psi_{xy}\right)}_{P}+a_{P}^{\Psi_{xy}\;u}u_{P}+a_{P}^{\Psi_{xy}\;v}v_{P}+\sum_{F=NB(P)}a_{F}^{\Psi_{xy}\;\Psi_{xy}}{\left(\Psi_{xy}\right)}_{F}+\\ & +\sum_{F=NB(P)}a_{F}^{\Psi_{xy}\;u}u_{F}+\sum_{F=NB(P)}a_{F}^{\Psi_{xy}\;v}v_{F}=b_{P}^{\Psi_{xy}}, \end{array} & \\ \begin{array}{cc} & a_{P}^{\Psi_{yy}\;\Psi_{yy}}{\left(\Psi_{yy}\right)}_{P}+a_{P}^{\Psi_{yy}\;u}u_{P}+a_{P}^{\Psi_{yy}\;v}v_{P}+\sum_{F=NB(P)}a_{F}^{\Psi_{yy}\;\Psi_{yy}}{\left(\Psi_{yy}\right)}_{F}+\\ & +\sum_{F=NB(P)}a_{F}^{\Psi_{yy}\;u}u_{F}+\sum_{F=NB(P)}a_{F}^{\Psi_{yy}\;v}v_{F}=b_{P}^{\Psi_{yy}}, \end{array} \end{array}\right. \end{array}$$

where $a_{P}^{\Psi_{ij}\phi }$ and $a_{F}^{\Psi_{ij}\phi }$ are the owner and neighbor coefficients in the discretized log-conformation tensor constitutive equations representing the tensor component $\Psi_{ij}$ and the variable ϕ interactions, respectively, and $b_{P}^{\Psi ij}$ is the source term.

Again, for the sake of conciseness, the discretization of the rate of change $\frac{\partial \Psi_{ij}}{\partial t}$ and advective $u_{k}\frac{\partial (\Psi_{ij})}{\partial x_{k}}$ terms in Equation (32) are not detailed here because similar discretizations were performed for the polymeric stress-tensor ${\left(\tau_{E}\right)}_{ij}$ (see previous work [28]).

Regarding the commutator term $\left[\Psi_{ij},\Omega_{ij}\right]$, we can write the following expanded form:

(36) $$\begin{array}{c} \left[\Psi_{ij},\Omega_{ij}\right]=\frac{1}{2}\left(\Psi_{ik}\frac{\partial u_{k}}{\partial x_{j}}-\Psi_{ik}\frac{\partial u_{j}}{\partial x_{k}}-\frac{\partial u_{i}}{\partial x_{k}}\Psi_{kj}+\frac{\partial u_{k}}{\partial x_{i}}\Psi_{kj}\right), \end{array}$$

and, subsequently, we can use Taylor approximations such as

(37) $$\begin{array}{c} \begin{array}{cc} & -\frac{1}{2}\left(\Psi_{ik}\frac{\partial u_{j}}{\partial x_{k}}+\frac{\partial u_{i}}{\partial x_{k}}\Psi_{kj}\right)\\ \approx & +\frac{1}{2}\left[\Psi_{ik}^{0}{\left(\frac{\partial u_{j}}{\partial x_{k}}\right)}^{0}+{\left(\frac{\partial u_{i}}{\partial x_{k}}\right)}^{0}\Psi_{kj}^{0}\right]-\frac{1}{2}\left[\Psi_{ik}{\left(\frac{\partial u_{j}}{\partial x_{k}}\right)}^{0}+{\left(\frac{\partial u_{i}}{\partial x_{k}}\right)}^{0}\Psi_{kj}\right]\\ & -\frac{1}{2}\left[\Psi_{ik}^{0}\frac{\partial u_{j}}{\partial x_{k}}+\frac{\partial u_{i}}{\partial x_{k}}\Psi_{kj}^{0}\right], \end{array} \end{array}$$

and

(38) $$\begin{array}{c} \begin{array}{cc} & +\frac{1}{2}\left(\Psi_{ik}\frac{\partial u_{k}}{\partial x_{j}}+\frac{\partial u_{k}}{\partial x_{i}}\Psi_{kj}\right)\\ \approx & -\frac{1}{2}\left[\Psi_{ik}^{0}{\left(\frac{\partial u_{k}}{\partial x_{j}}\right)}^{0}+{\left(\frac{\partial u_{k}}{\partial x_{i}}\right)}^{0}\Psi_{kj}^{0}\right]+\frac{1}{2}\left[\Psi_{ik}{\left(\frac{\partial u_{k}}{\partial x_{j}}\right)}^{0}+{\left(\frac{\partial u_{k}}{\partial x_{i}}\right)}^{0}\Psi_{kj}\right]\\ & +\frac{1}{2}\left[\Psi_{ik}^{0}\frac{\partial u_{k}}{\partial x_{j}}+\frac{\partial u_{k}}{\partial x_{i}}\Psi_{kj}^{0}\right]. \end{array} \end{array}$$

Starting with the terms in Equation (37), the negative commutator terms, the first contribution is explicit, being given by

(39) $$\begin{array}{c} {\left(b_{P}^{\Psi_{ij}}\right)}_{neg}=-\frac{1}{2}V_{P}\left[\Psi_{ik}^{0}{\left(\frac{\partial u_{j}}{\partial x_{k}}\right)}^{0}+{\left(\frac{\partial u_{i}}{\partial x_{k}}\right)}^{0}\Psi_{kj}^{0}\right]. \end{array}$$

The second contribution is implicit in $\Psi_{ij}$ and explicit in $\frac{\partial u_{i}}{\partial x_{j}}$, being given by

(40a) $$\begin{array}{c} {\left(a_{P}^{\Psi_{ij}\Psi_{ik}}\right)}_{neg,1}=-\frac{1}{2}V_{P}{\left(\frac{\partial u_{j}}{\partial x_{k}}\right)}^{0}, \end{array}$$

(40b) $$\begin{array}{c} {\left(a_{P}^{\Psi_{ij}\Psi_{kj}}\right)}_{neg,2}=-\frac{1}{2}V_{P}{\left(\frac{\partial u_{i}}{\partial x_{k}}\right)}^{0}. \end{array}$$

The third contribution is implicit in $\frac{\partial u_{i}}{\partial x_{j}}$ and explicit in $\Psi_{ij}$; therefore, we need an implicit discretization of the gradient operator for velocity, which requires the integration by parts of this term, such as:

(41) $$\begin{array}{c} \begin{array}{cc} & \int_{V_{P}}\left(\Psi_{ik}^{0}\frac{\partial u_{j}}{\partial x_{k}}+\frac{\partial u_{i}}{\partial x_{k}}\Psi_{kj}^{0}\right)dV_{P}\\ = & \oint_{S}n_{k}\left(\Psi_{ik}^{0}u_{j}+u_{i}\Psi_{kj}^{0}\right)dS-\int_{V_{P}}\left[{\left(\frac{\partial \Psi_{ik}}{\partial x_{k}}\right)}^{0}u_{j}+u_{i}{\left(\frac{\partial \Psi_{kj}}{\partial x_{k}}\right)}^{0}\right]dV_{P}. \end{array} \end{array}$$

Applying the Gauss divergence theorem, the discretized form of the terms on the right-hand side of Equation (41) is

(42) $$\begin{array}{c} \sum_{f=nb(P)}{\left[s_{k}\left(\Psi_{ik}^{0}u_{j}+u_{i}\Psi_{kj}^{0}\right)\right]}_{f}-V_{P}\left[{\left(\frac{\partial \Psi_{ik}}{\partial x_{k}}\right)}^{0}u_{j}+u_{i}{\left(\frac{\partial \Psi_{kj}}{\partial x_{k}}\right)}^{0}\right]. \end{array}$$

Using linear weighted interpolation, we can write the contributions of the third term as:

(43a) $$\begin{array}{cc} \; & {\left(a_{F}^{\Psi_{ij}u_{j}}\right)}_{neg,1}=-\frac{1}{2}{\left(s_{k}\Psi_{ik}^{0}\right)}_{f}\left(1-w_{f}\right), \end{array}$$

(43b) $$\begin{array}{cc} \; & {\left(a_{P}^{\Psi_{ij}u_{j}}\right)}_{neg,1}=-\frac{1}{2}\left[\sum_{f=nb(P)}{\left(s_{k}\Psi_{ik}^{0}\right)}_{f}w_{f}-V_{P}{\left(\frac{\partial \Psi_{ik}}{\partial x_{k}}\right)}^{0}\right], \end{array}$$

(43c) $$\begin{array}{cc} \; & {\left(a_{F}^{\Psi_{ij}u_{i}}\right)}_{neg,2}=-\frac{1}{2}{\left(\Psi_{kb}^{0}s_{k}\right)}_{f}\left(1-w_{f}\right), \end{array}$$

(43d) $$\begin{array}{cc} \; & {\left(a_{P}^{\Psi_{ij}u_{i}}\right)}_{neg,2}=-\frac{1}{2}\left[\sum_{f=nb(P)}{\left(s_{k}\Psi_{kj}^{0}\right)}_{f}w_{f}-V_{P}{\left(\frac{\partial \Psi_{kj}}{\partial x_{k}}\right)}^{0}\right]. \end{array}$$

Following the same reasoning as given above, the terms in Equation (38), the positive commutator terms, generate the following coefficients:

(44) $$\begin{array}{c} {\left(b_{P}^{\Psi_{ij}}\right)}_{pos}=\frac{1}{2}V_{P}\left[\Psi_{ik}^{0}{\left(\frac{\partial u_{k}}{\partial x_{j}}\right)}^{0}+{\left(\frac{\partial u_{k}}{\partial x_{i}}\right)}^{0}\Psi_{kj}^{0}\right], \end{array}$$

(45a) $$\begin{array}{c} {\left(a_{P}^{\Psi_{ij}\Psi_{ik}}\right)}_{pos,1}=\frac{1}{2}V_{P}{\left(\frac{\partial u_{k}}{\partial x_{j}}\right)}^{0}, \end{array}$$

(45b) $$\begin{array}{c} {\left(a_{P}^{\Psi_{ij}\Psi_{kj}}\right)}_{pos,2}=\frac{1}{2}V_{P}{\left(\frac{\partial u_{k}}{\partial x_{i}}\right)}^{0}, \end{array}$$

(46a) $$\begin{array}{cc} \; & {\left(a_{F}^{\Psi_{ij}u_{k}}\right)}_{pos,1}=\frac{1}{2}{\left(s_{j}\Psi_{ik}^{0}\right)}_{f}\left(1-w_{f}\right), \end{array}$$

(46b) $$\begin{array}{cc} \; & {\left(a_{P}^{\Psi_{ij}u_{k}}\right)}_{pos,1}=\frac{1}{2}\left[\sum_{f=nb(P)}{\left(s_{j}\Psi_{ik}^{0}\right)}_{f}w_{f}-V_{P}{\left(\frac{\partial \Psi_{ik}}{\partial x_{j}}\right)}^{0}\right], \end{array}$$

(46c) $$\begin{array}{cc} \; & {\left(a_{F}^{\Psi_{ij}u_{k}}\right)}_{pos,2}=\frac{1}{2}{\left(s_{i}\Psi_{kj}^{0}\right)}_{f}\left(1-w_{f}\right), \end{array}$$

(46d) $$\begin{array}{cc} \; & {\left(a_{P}^{\Psi_{ij}u_{k}}\right)}_{pos,2}=\frac{1}{2}\left[\sum_{f=nb(P)}{\left(s_{i}\Psi_{kj}^{0}\right)}_{f}w_{f}-V_{P}{\left(\frac{\partial \Psi_{kj}}{\partial x_{i}}\right)}^{0}\right]. \end{array}$$

Lastly, we implicitly discretize the adjoint operator $f{\left(\mathrm{ad}\left(\Psi \right)\right)}_{ijkl}D_{kl}$ by considering the following Taylor approximation [39]

(47) $$\begin{array}{c} \begin{array}{cc} & f{\left(\mathrm{ad}\left(\Psi \right)\right)}_{ijkl}D_{kl}\\ \approx & f{\left(\mathrm{ad}\left(\Psi^{0}\right)\right)}_{ijkl}D_{kl}^{0}+\sum_{kl}\frac{\partial \left(f{\left(\mathrm{ad}\left(\Psi^{0}\right)\right)}_{iqrj}D_{qr}^{0}\right)}{\partial \Psi_{kl}}\left(\Psi_{kl}-\Psi_{kl}^{0}\right)\\ & +\sum_{kl}\frac{\partial \left(f{\left(\mathrm{ad}\left(\Psi^{0}\right)\right)}_{iqrj}D_{qr}^{0}\right)}{\partial D_{kl}}\left(D_{kl}-D_{kl}^{0}\right), \end{array} \end{array}$$

where $\frac{\partial \left(f{\left(\mathrm{ad}\left(\Psi^{0}\right)\right)}_{iqrj}\;D_{qr}^{0}\right)}{\partial D_{kl}}$ is the derivative of the adjoint operator with respect to $D_{kl}$, given by [38]

(48) $$\begin{array}{c} \begin{array}{cc} & \frac{\partial \left(f{\left(\mathrm{ad}\left(\Psi^{0}\right)\right)}_{iqrj}D_{qr}\right)}{\partial D_{kl}}\\ = & f{\left(\mathrm{ad}\left(\Psi^{0}\right)\right)}_{ijkl}\\ = & \sum_{m,n=1}^{d}f\left(\lambda_{m}-\lambda_{n}\right)p_{ik}^{m}p_{lj}^{n}, \end{array} \end{array}$$

and $\frac{\partial \left(f{\left(\mathrm{ad}\left(\Psi^{0}\right)\right)}_{iqrj}\;D_{qr}^{0}\right)}{\partial \Psi_{kl}}$ is the derivative of the adjoint operator with respect to $\Psi_{kl}$, given by [49]

(49) $$\begin{array}{c} \begin{array}{cc} & \frac{\partial \left(f{\left(\mathrm{ad}\left(\Psi^{0}\right)\right)}_{iqrj}D_{qr}^{0}\right)}{\partial \Psi_{kl}}\\ = & \sum_{m,n,o=1}^{d}\frac{f\left(\lambda_{m}-\lambda_{o}\right)-f\left(\lambda_{n}-\lambda_{o}\right)}{\lambda_{m}-\lambda_{n}}\left(p_{ik}^{m}p_{lq}^{n}D_{qr}p_{rj}^{o}+p_{iq}^{o}D_{qr}p_{rk}^{n}p_{lj}^{m}\right). \end{array} \end{array}$$

Note that, if $\lambda_{m}$ is equal to $\lambda_{n}$, then the denominator of Equation (49) needs to be replaced by $f^{\prime }\left(\lambda_{m}-\lambda_{n}\right)$ [38].

Subsequently, we integrate the adjoint term over a control volume $V_{P}$ as follows:

(50) $$\begin{array}{c} \begin{array}{cc} & \int_{V_{P}}2f{\left(\mathrm{ad}\left(\Psi \right)\right)}_{ijkl}D_{kl}dV_{P}\\ \approx & \int_{V_{P}}2f{\left(\mathrm{ad}\left(\Psi^{0}\right)\right)}_{ijkl}D_{kl}^{0}dV_{P}+\int_{V_{P}}2{\left[\frac{\partial \left(f{\left(\mathrm{ad}\left(\Psi \right)\right)}_{iqrj}D_{qr}\right)}{\partial \Psi_{kl}}\right]}^{0}\Psi_{kl}dV_{P}\\ & -\int_{V_{P}}2{\left[\frac{\partial \left(f{\left(\mathrm{ad}\left(\Psi \right)\right)}_{iqrj}D_{qr}\right)}{\partial \Psi_{kl}}\right]}^{0}\Psi_{kl}^{0}dV_{P}+\int_{V_{P}}2{\left[\frac{\partial \left(f{\left(\mathrm{ad}\left(\Psi \right)\right)}_{iqrj}D_{qr}\right)}{\partial D_{kl}}\right]}^{0}D_{kl}dV_{P}\\ & -\int_{V_{P}}2{\left[\frac{\partial \left(f{\left(\mathrm{ad}\left(\Psi \right)\right)}_{iqrj}D_{qr}\right)}{\partial D_{kl}}\right]}^{0}D_{kl}^{0}dV_{P}, \end{array} \end{array}$$

and substituting $D_{kl}=\frac{1}{2}\left(\frac{\partial u_{k}}{\partial x_{l}}+\frac{\partial u_{l}}{\partial x_{k}}\right)$ in Equation (50), we obtain

(51) $$\begin{array}{c} \begin{array}{cc} & \int_{V_{P}}2f{\left(\mathrm{ad}\left(\Psi \right)\right)}_{ijkl}D_{kl}dV_{P}\\ \approx & \int_{V_{P}}2f{\left(\mathrm{ad}\left(\Psi^{0}\right)\right)}_{ijkl}D_{kl}^{0}dV_{P}+\int_{V_{P}}2{\left[\frac{\partial \left(f{\left(\mathrm{ad}\left(\Psi \right)\right)}_{iqrj}D_{qr}\right)}{\partial \Psi_{kl}}\right]}^{0}\Psi_{kl}dV_{P}\\ & -\int_{V_{P}}2{\left[\frac{\partial \left(f{\left(\mathrm{ad}\left(\Psi \right)\right)}_{iqrj}D_{qr}\right)}{\partial \Psi_{kl}}\right]}^{0}\Psi_{kl}^{0}dV_{P}\\ & +\int_{V_{P}}{\left[\frac{\partial \left(f{\left(\mathrm{ad}\left(\Psi \right)\right)}_{iqrj}D_{qr}\right)}{\partial D_{kl}}\right]}^{0}\left(\frac{\partial u_{k}}{\partial x_{l}}+\frac{\partial u_{l}}{\partial x_{k}}\right)dV_{P}\\ & -\int_{V_{P}}2{\left[\frac{\partial \left(f{\left(\mathrm{ad}\left(\Psi \right)\right)}_{iqrj}D_{qr}\right)}{\partial D_{kl}}\right]}^{0}D_{kl}^{0}dV_{P}. \end{array} \end{array}$$

As the fourth term on the right hand side of Equation (51) contains implicit velocity gradients, we employ integration by parts to linearize them, obtaining

(52) $$\begin{array}{c} \begin{array}{cc} & \int_{V_{P}}2f{\left(\mathrm{ad}\left(\Psi \right)\right)}_{ijkl}D_{kl}dV_{P}\\ \approx & \int_{V_{P}}2f{\left(\mathrm{ad}\left(\Psi^{0}\right)\right)}_{ijkl}D_{kl}^{0}dV_{P}+\int_{V_{P}}2{\left[\frac{\partial \left(f{\left(\mathrm{ad}\left(\Psi \right)\right)}_{iqrj}D_{qr}\right)}{\partial \Psi_{kl}}\right]}^{0}\Psi_{kl}dV_{P}\\ & -\int_{V_{P}}2{\left[\frac{\partial \left(f{\left(\mathrm{ad}\left(\Psi \right)\right)}_{iqrj}D_{qr}\right)}{\partial \Psi_{kl}}\right]}^{0}\Psi_{kl}^{0}dV_{P}\\ & +\oint_{S}{\left[\frac{\partial \left(f{\left(\mathrm{ad}\left(\Psi \right)\right)}_{iqrj}D_{qr}\right)}{\partial D_{kl}}\right]}^{0}\left(u_{k}n_{l}+u_{l}n_{k}\right)dS\\ & -\int_{V_{P}}\left[\frac{\partial {\left[\frac{\partial \left(f{(\mathrm{ad}\left(\Psi \right))\;}_{iqrj}\;D_{qr}\right)}{\partial D_{kl}}\right]}^{0}}{\partial x_{l}}u_{k}+\frac{\partial {\left[\frac{\partial \left(f{(\mathrm{ad}\left(\Psi \right))\;}_{iqrj}\;D_{qr}\right)}{\partial D_{kl}}\right]}^{0}}{\partial x_{k}}u_{l}\right]dV_{P}\\ & -\int_{V_{P}}2{\left[\frac{\partial \left(f{\left(\mathrm{ad}\left(\Psi \right)\right)}_{iqrj}D_{qr}\right)}{\partial D_{kl}}\right]}^{0}D_{kl}^{0}dV_{P}. \end{array} \end{array}$$

Thus, the discretized form of Equation (52) can then be written as:

(53) $$\begin{array}{c} \begin{array}{cc} & \int_{V_{P}}2f{\left(\mathrm{ad}\left(\Psi \right)\right)}_{ijkl}D_{kl}dV_{P}\\ \approx & 2f{\left(\mathrm{ad}\left(\Psi^{0}\right)\right)}_{ijkl}D_{kl}^{0}V_{P}+2{\left[\frac{\partial \left(f{\left(\mathrm{ad}\left(\Psi \right)\right)}_{iqrj}D_{qr}\right)}{\partial \Psi_{kl}}\right]}^{0}\Psi_{kl}V_{P}\\ & -2{\left[\frac{\partial \left(f{\left(\mathrm{ad}\left(\Psi \right)\right)}_{iqrj}D_{qr}\right)}{\partial \Psi_{kl}}\right]}^{0}\Psi_{kl}^{0}V_{P}+\sum_{f=nb(P)}{\left[{\left[\frac{\partial \left(f{\left(\mathrm{ad}\left(\Psi \right)\right)}_{iqrj}D_{qr}\right)}{\partial D_{kl}}\right]}^{0}\left(u_{k}s_{l}+u_{l}s_{k}\right)\right]}_{f}\\ & -\left[\frac{\partial {\left[\frac{\partial \left(f{(\mathrm{ad}\left(\Psi \right))\;}_{iqrj}\;D_{qr}\right)}{\partial D_{kl}}\right]}^{0}}{\partial x_{l}}u_{k}+\frac{\partial {\left[\frac{\partial \left(f{(\mathrm{ad}\left(\Psi \right))\;}_{iqrj}\;D_{qr}\right)}{\partial D_{kl}}\right]}^{0}}{\partial x_{k}}u_{l}\right]V_{P}\\ & -2{\left[\frac{\partial \left(f{\left(\mathrm{ad}\left(\Psi \right)\right)}_{iqrj}D_{qr}\right)}{\partial D_{kl}}\right]}^{0}D_{kl}^{0}V_{P}. \end{array} \end{array}$$

The contributions of the adjoint term for the log-conformation tensor constitutive equations, using linear weighted interpolation, read as

(54a) $$\begin{array}{cc} \; & {\left(a_{P}^{\Psi_{ij}\Psi_{kl}}\right)}_{adj}=2{\left[\frac{\partial \left(f{\left(\mathrm{ad}\left(\Psi \right)\right)}_{iqrj}D_{qr}\right)}{\partial \Psi_{kl}}\right]}^{0}V_{P}, \end{array}$$

(54b) $$\begin{array}{cc} \; & {\left(a_{F}^{\Psi_{ij}u_{k}}\right)}_{adj}={\left[{\left[\frac{\partial \left(f{\left(\mathrm{ad}\left(\Psi \right)\right)}_{iqrj}D_{qr}\right)}{\partial D_{kl}}\right]}^{0}s_{l}\right]}_{f}\left(1-w_{f}\right), \end{array}$$

(54c) $$\begin{array}{cc} \; & {\left(a_{P}^{\Psi_{ij}u_{k}}\right)}_{adj}=\sum_{f=nb(P)}{\left[{\left[\frac{\partial \left(f{\left(\mathrm{ad}\left(\Psi \right)\right)}_{iqrj}D_{qr}\right)}{\partial D_{kl}}\right]}^{0}s_{l}\right]}_{f}w_{f}-\frac{\partial {\left[\frac{\partial \left(f{(\mathrm{ad}\left(\Psi \right))\;}_{iqrj}\;D_{qr}\right)}{\partial D_{kl}}\right]}^{0}}{\partial x_{l}}V_{P}, \end{array}$$

(54d) $$\begin{array}{cc} \; & {\left(a_{F}^{\Psi_{ij}u_{l}}\right)}_{adj}={\left[{\left[\frac{\partial \left(f{\left(\mathrm{ad}\left(\Psi \right)\right)}_{iqrj}D_{qr}\right)}{\partial D_{kl}}\right]}^{0}s_{k}\right]}_{f}\left(1-w_{f}\right), \end{array}$$

(54e) $$\begin{array}{cc} \; & {\left(a_{P}^{\Psi_{ij}u_{l}}\right)}_{adj}=\sum_{f=nb(P)}{\left[{\left[\frac{\partial \left(f{\left(\mathrm{ad}\left(\Psi \right)\right)}_{iqrj}D_{qr}\right)}{\partial D_{kl}}\right]}^{0}s_{k}\right]}_{f}w_{f}-\frac{\partial {\left[\frac{\partial \left(f{(\mathrm{ad}\left(\Psi \right))\;}_{iqrj}\;D_{qr}\right)}{\partial D_{kl}}\right]}^{0}}{\partial x_{k}}V_{P}, \end{array}$$

(54f) $$\begin{array}{cc} {\left(b_{P}^{\Psi_{ij}}\right)}_{adj}= & 2f{\left(\mathrm{ad}\left(\Psi^{0}\right)\right)}_{ijkl}D_{kl}^{0}V_{P}-2{\left[\frac{\partial \left(f{\left(\mathrm{ad}\left(\Psi \right)\right)}_{iqrj}D_{qr}\right)}{\partial \Psi_{kl}}\right]}^{0}\Psi_{kl}^{0}V_{P}\\ & -2{\left[\frac{\partial \left(f{\left(\mathrm{ad}\left(\Psi \right)\right)}_{iqrj}D_{qr}\right)}{\partial D_{kl}}\right]}^{0}D_{kl}^{0}V_{P}. \end{array}$$

Again, it should be noted that the total contribution of the owner and neighbor coefficients related to the log-conformation tensor components’ interactions is given by the sum of the log-conformation tensor, rate of change, advection, commutator and adjoint terms. In addition, the total contribution for the explicit coefficient related to the log-conformation tensor constitutive equations is given by the sum of the rate of change, commutator and adjoint terms.

### 3.4. Discretization of the Equation for Conservation of Energy

The discretization starts by integrating the equation for the conservation of energy (Equation (13)) over a general control volume $V_{P}$, to yield

(55) $$\begin{array}{c} \begin{array}{cc} & \rho C_{p}\left(\int_{V_{P}}\frac{\partial T}{\partial t}dV_{P}+\int_{V_{P}}u_{i}\frac{\partial T}{\partial x_{i}}dV_{P}\right)-\int_{V_{P}}k\frac{\partial^{2}T}{\partial x_{i}^{2}}dV_{P}\\ = & \int_{V_{P}}\left[{\left(\tau_{N}\right)}_{ij}D_{ji}+\frac{\eta_{E}\left(T\right)}{\lambda (T)}\left(\alpha {\left(e^{\Psi }-I\right)}_{ij}D_{ji}+\left(1-\alpha \right)\frac{{\left(e^{\Psi }-I\right)}_{ii}}{2\bar{\lambda }\left(T\right)}\right)\right]dV_{P}. \end{array} \end{array}$$

Using the Gauss divergence theorem, the volume integrals of the advection and diffusion terms in Equation (55) are transformed into surface integrals as:

(56) $$\begin{array}{c} \begin{array}{cc} & \rho C_{p}\left(\int_{V_{P}}\frac{\partial T}{\partial t}dV_{P}+\oint_{S}n_{i}u_{i}TdS\right)-\oint_{S}n_{i}\left(k\frac{\partial T}{\partial x_{i}}\right)dS\\ = & \int_{V_{P}}\left[{\left(\tau_{N}\right)}_{ij}D_{ji}+\frac{\eta_{E}\left(T\right)}{\lambda (T)}\left(\alpha {\left(e^{\Psi }-I\right)}_{ij}D_{ji}+\left(1-\alpha \right)\frac{{\left(e^{\Psi }-I\right)}_{ii}}{2\bar{\lambda }\left(T\right)}\right)\right]dV_{P}. \end{array} \end{array}$$

The semi-discretized equation for the conservation of energy is obtained by evaluating the surface integrals using a second-order integration scheme and approximating the rate of change term with a backward implicit Euler scheme, such as

(57) $$\begin{array}{c} \begin{array}{cc} & V_{P}\rho_{P}{\left(C_{p}\right)}_{P}\frac{T_{P}-T_{P}^{0}}{\Delta t}+\sum_{f=nb(P)}{\left(s_{i}\rho C_{p}u_{i}T\right)}_{f}-\sum_{f=nb(P)}{\left(s_{i}k\frac{\partial T}{\partial x_{i}}\right)}_{f}\\ = & V_{P}\left[{\left(\tau_{N}\right)}_{ij}D_{ji}+\frac{\eta_{E}\left(T\right)}{\lambda (T)}\left(\alpha {\left(e^{\Psi }-I\right)}_{ij}D_{ji}+\left(1-\alpha \right)\frac{{\left(e^{\Psi }-I\right)}_{ii}}{2\bar{\lambda }\left(T\right)}\right)\right]. \end{array} \end{array}$$

This leads to the algebraic equation for the energy balance with the following form:

(58) $$\begin{array}{c} a_{P}^{TT}T_{P}+a_{P}^{Tu}u_{P}+a_{P}^{Tv}v_{P}+\sum_{F=NB(P)}a_{F}^{TT}T_{F}+\sum_{F=NB(P)}a_{F}^{Tu}u_{F}+\sum_{F=NB(P)}a_{F}^{Tv}v_{F}=b_{P}^{T}, \end{array}$$

where $a_{P}^{T\phi }$ and $a_{F}^{T\phi }$ are the owner and neighbor coefficients in the discretized conservation of energy equation, representing the temperature *T* and the variable ϕ interactions, respectively, and $b_{P}^{T}$ is the source term.

The rate of change (rchg) term in Equation (57) (first term), contributes to both the diagonal of the system of equations and to the explicit term as:

(59a) $$\begin{array}{cc} \; & a_{P,rchg}^{TT}=\frac{V_{P}\;\rho_{P}\;{\left(C_{p}\right)}_{P}}{\Delta t}, \end{array}$$

(59b) $$\begin{array}{cc} \; & b_{P,rchg}^{T}=\frac{V_{P}\;\rho_{P}\;{\left(C_{p}\right)}_{P}T_{P}^{0}}{\Delta t}. \end{array}$$

Then, and in the framework of the FVM, the advection term in Equation (57) (second term) is linearized by computing the mass flow rate at control-volume face *f*$\left({\dot{m}}_{f}={\left(s_{i}\rho u_{i}\right)}_{f}\right)$ using the previous iteration values. Here, we used the UDS differentiating scheme to approximate the advection term. However, many high-order schemes could be used, such as the MINMOD or CUBISTA schemes. For the sake of readability, the discretization procedure will be presented for the UDS scheme, but it is important to stress that the methodology is independent of the adopted discretization scheme. In this way, the coefficients of the advection (adv) term contribution for the conservation of energy equation are given by:

(60a) $$\begin{array}{cc} \; & a_{F,adv}^{Tu}=a_{F,adv}^{Tv}={\left(C_{p}\right)}_{f}\max\left({\dot{m}}_{f},\;0\right), \end{array}$$

(60b) $$\begin{array}{cc} \; & a_{P,adv}^{Tu}=-\sum_{F=NB(P)}a_{F,adv}^{Tu},\;a_{P,adv}^{Tv}=-\sum_{F=NB(P)}a_{F,adv}^{Tv}, \end{array}$$

where the superscript Tϕ means the influence of the ϕ variable in the *T* energy equation. The term $\max({\dot{m}}_{f},\;0)$ represents the maximum of ${\dot{m}}_{f}$ and 0, where the mass flux is positive if it goes from owner to neighbor cells, i.e., leaves the control-volume $V_{P}$.

The implicit diffusion (idiff) contribution, third term of Equation (57), is discretized, taking a linear profile (see page 86 of [48]) as

(61) $$\begin{array}{c} {\left(s_{i}k\frac{\partial T}{\partial x_{i}}\right)}_{f}=k_{f}\frac{{\left(s_{i}s_{i}\right)}_{f}}{{\left(d_{PF}\right)}_{i}{\left(s_{i}\right)}_{f}}\left(T_{F}-T_{P}\right), \end{array}$$

where ${\left(d_{PF}\right)}_{i}$ is the vector joining the centroids *P* and *F* (see Figure 1). The coefficients of the implicit diffusion term for the conservation of energy equation are given by

(62a) $$\begin{array}{cc} \; & a_{F,idiff}^{TT}=-k_{f}\frac{{\left(s_{i}s_{i}\right)}_{f}}{{\left(d_{PF}\right)}_{i}{\left(s_{i}\right)}_{f}}, \end{array}$$

(62b) $$\begin{array}{cc} \; & a_{P,idiff}^{TT}=-\sum_{F=NB(P)}a_{F,idiff}^{TT}. \end{array}$$

Finally, the coefficients of the explicit term contribution (right-hand side of Equation (57)) for the conservation of energy equation are given by

(63) $$\begin{array}{c} \begin{array}{cc} & b_{P,source}^{T}=V_{P}\left[{\left(\tau_{N}\right)}_{ij}D_{ji}+\frac{\eta_{E}\left(T\right)}{\lambda (T)}\left(\alpha {\left(e^{\Psi }-I\right)}_{ij}D_{ji}+\left(1-\alpha \right)\frac{{\left(e^{\Psi }-I\right)}_{ii}}{2\bar{\lambda }\left(T\right)}\right)\right]. \end{array} \end{array}$$

### 3.5. Block-Coupled Algorithm

Combining the discretized conservation of linear momentum (Equation (17)), conservation of mass (Equation (27)), log-conformation tensor (Equation (35)) and conservation of energy equations (Equation (58)), the following linear system of equations, written in matrix form, is obtained for each control volume:

(64) $$\begin{array}{c} \begin{array}{cc} & \left[\begin{array}{ccccccc} a_{P}^{uu} & a_{P}^{uv} & a_{P}^{up} & a_{P}^{u\Psi_{xx}} & a_{P}^{u\Psi_{xy}} & a_{P}^{u\Psi_{yy}} & a_{P}^{uT}\\ a_{P}^{vu} & a_{P}^{vv} & a_{P}^{vp} & a_{P}^{v\Psi_{xx}} & a_{P}^{v\Psi_{xy}} & a_{P}^{v\Psi_{yy}} & a_{P}^{vT}\\ a_{P}^{pu} & a_{P}^{pv} & a_{P}^{pp} & a_{P}^{p\Psi_{xx}} & a_{P}^{p\Psi_{xy}} & a_{P}^{p\Psi_{yy}} & a_{P}^{pT}\\ a_{P}^{\Psi_{xx}u} & a_{P}^{\Psi_{xx}\;v} & a_{P}^{\Psi_{xx}\;p} & a_{P}^{\Psi_{xx}\;\Psi_{xx}} & a_{P}^{\Psi_{xx}\;\Psi_{xy}} & a_{P}^{\Psi_{xx}\;\Psi_{yy}} & a_{P}^{\Psi_{xx}\;T}\\ a_{P}^{\Psi_{xy}\;u} & a_{P}^{\Psi_{xy}\;v} & a_{P}^{\Psi_{xy}\;p} & a_{P}^{\Psi_{xy}\;\Psi_{xx}} & a_{P}^{\Psi_{xy}\;\Psi_{xy}} & a_{P}^{\Psi_{xy}\;\Psi_{yy}} & a_{P}^{\Psi_{xy}\;T}\\ a_{P}^{\Psi_{yy}\;u} & a_{P}^{\Psi_{yy}\;v} & a_{P}^{\Psi_{yy}\;p} & a_{P}^{\Psi_{yy}\;\Psi_{xx}} & a_{P}^{\Psi_{yy}\;\Psi_{xy}} & a_{P}^{\Psi_{yy}\;\Psi_{yy}} & a_{P}^{\Psi_{yy}\;T}\\ a_{P}^{Tu} & a_{P}^{Tv} & a_{P}^{Tp} & a_{P}^{T\Psi_{xx}} & a_{P}^{T\Psi_{xy}} & a_{P}^{T\Psi_{yy}} & a_{P}^{TT} \end{array}\right]\left[\begin{array}{c} u_{P}\\ v_{P}\\ p_{P}\\ {\left(\Psi_{xx}\right)}_{P}\\ {\left(\Psi_{xy}\right)}_{P}\\ {\left(\Psi_{yy}\right)}_{P}\\ T_{P} \end{array}\right]+\\ & +\sum_{F=nb(P)}\left[\begin{array}{ccccccc} a_{F}^{uu} & a_{F}^{uv} & a_{F}^{up} & a_{F}^{u\Psi_{xx}} & a_{F}^{u\Psi_{xy}} & a_{F}^{u\Psi_{yy}} & a_{F}^{uT}\\ a_{F}^{vu} & a_{F}^{vv} & a_{F}^{vp} & a_{F}^{v\Psi_{xx}} & a_{F}^{v\Psi_{xy}} & a_{F}^{v\Psi_{yy}} & a_{F}^{vT}\\ a_{F}^{pu} & a_{F}^{pv} & a_{F}^{pp} & a_{F}^{p\Psi_{xx}} & a_{F}^{p\Psi_{xy}} & a_{F}^{p\Psi_{yy}} & a_{F}^{pT}\\ a_{F}^{\Psi_{xx}\;u} & a_{F}^{\Psi_{xx}\;v} & a_{F}^{\Psi_{xx}\;p} & a_{F}^{\Psi_{xx}\;\Psi_{xx}} & a_{F}^{\Psi_{xx}\;\Psi_{xy}} & a_{F}^{\Psi_{xx}\;\Psi_{yy}} & a_{F}^{\Psi_{xx}\;T}\\ a_{F}^{\Psi_{xy}\;u} & a_{F}^{\Psi_{xy}\;v} & a_{F}^{\Psi_{xy}\;p} & a_{F}^{\Psi_{xy}\;\Psi_{xx}} & a_{F}^{\Psi_{xy}\;\Psi_{xy}} & a_{F}^{\Psi_{xy}\;\Psi_{yy}} & a_{F}^{\Psi_{xy}\;T}\\ a_{F}^{\Psi_{yy}\;u} & a_{F}^{\Psi_{yy}\;v} & a_{F}^{\Psi_{yy}\;p} & a_{F}^{\Psi_{yy}\;\Psi_{xx}} & a_{F}^{\Psi_{yy}\;\Psi_{xy}} & a_{F}^{\Psi_{yy}\;\Psi_{yy}} & a_{F}^{\Psi_{yy}\;T}\\ a_{F}^{Tu} & a_{F}^{Tv} & a_{F}^{Tp} & a_{F}^{T\Psi_{xx}} & a_{F}^{T\Psi_{xy}} & a_{F}^{T\Psi_{yy}} & a_{F}^{TT} \end{array}\right]\left[\begin{array}{c} u_{F}\\ v_{F}\\ p_{F}\\ {\left(\Psi_{xx}\right)}_{F}\\ {\left(\Psi_{xy}\right)}_{F}\\ {\left(\Psi_{yy}\right)}_{F}\\ T_{F} \end{array}\right]=\left[\begin{array}{c} b_{P}^{u}\\ b_{P}^{v}\\ b_{P}^{p}\\ b_{P}^{\Psi_{xx}}\\ b_{P}^{\Psi_{xy}}\\ b_{P}^{\Psi_{yy}}\\ b_{P}^{T} \end{array}\right] \end{array} \end{array}$$

The linear systems (Equation (64)) obtained for each control volume of the computational domain are merged in a full system of equations, which can be written in the form AΦ=b where all variables $\Phi =(u_{i},\;p,\;\Psi_{ij},\;T)$ are solved simultaneously. In this procedure, all variables in the different equations are treated implicitly, which is expected to be advantageous to the stability of the overall calculation process. The fully implicit coupled algorithm can be summarized into the following steps:

1. Initialize the fluid variables with the latest known values $\left(u_{i}^{n},\;p^{n},\;\Psi_{ij}^{n},T^{n}\right)$.
2. Assemble the discretized equations for the conservation of linear momentum, conservation of mass, log-conformation tensor and conservation of energy (see Equations (17), (27), (35), (58)) and solve for $u_{i}$, *p*, $\Psi_{ij}$ and *T* (Equation (64)).
3. Iterate until convergence.

For the solution of the global system of discretized algebraic equations, it is fundamental that an efficient linear solver is used to obtain the best overall convergence. In this work, the iterative solver Bi-Conjugate Gradient Stabilized (BiCGStab) [50], combined with an LU preconditioner, was used to retrieve the solution of the global system of discretized algebraic equations (see detailed discussion in Pimenta and Alves [30]). The initial residual for each iteration is evaluated based on the current values of the field, before solving the block-coupled system. After each block solver linear iteration, the residual is re-evaluated (final residual). When the maximum number of linear iterations (in this work defined as 100) or the final residual falls below the solver absolute tolerance (set as $10^{-6}$), the block-coupled system current iteration stops and advances in time. All computations are performed on a computer with a 2.00-GHz 64 cores AMD EPYC 7662 CPU processor and 128 GB of RAM.

## 4. Results and Discussion

The validation of the newly-developed, fully implicit, block-coupled, non-isothermal, viscoelastic, log-conformation tensor-based algorithm was performed for the laminar, incompressible, non-isothermal viscoelastic flow of an Oldroyd-B fluid in an axisymmetric 4:1 sudden contraction geometry. For assessment purposes, the results computed with the newly-developed code were compared with the available data from the scientific literature [18].

### 4.1. Geometry, Meshes, and Initial and Boundary Conditions

**Figure 2:**
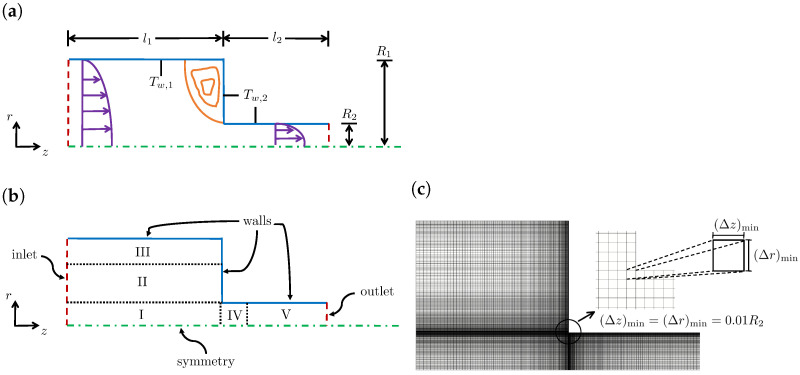
(**a**) Schematic of the 4:1 planar sudden contraction cross-section used to simulate the non-isothermal flow of a viscoelastic matrix fluid described by the Oldroyd-B constitutive model. The upstream length was $l_{1}$ and the downstream length was $l_{2}$. The upstream channel height was R1 and the downstream channel height was R2. The temperature at the upstream wall is $T_{w,1}$, while, for the downstream wall, the temperature was $T_{w,2}$. (**b**) The geometrical domain was divided into five blocks for mesh discretization. (**c**) A detailed view of the contraction area with the minimum normalized cell size at the corner ${\left(\Delta z\right)}_{\min}={\left(\Delta r\right)}_{\min}=0.01R_{2}$.

An axisymmetric 4:1 sudden contraction with ratio of the radii $R_{1}/R_{2}=4$ was chosen as test geometry (Figure 2a), because of the availability of numerical data in the literature [18]. The upstream length was $l_{1}=80R_{2}$ and the downstream length was $l_{2}=50R_{2}$. The downstream channel height was chosen as $R_{2}=0.0020604$ m.

The flow had a rotational symmetry that was normal to the rz−plane and, to save computational resources and reduce the CPU times, only half of the domain was considered. The characteristics of the meshes used to discretize the geometrical domain are presented in Table 1. The meshes employed in the current work (M1 and M2) resulted from a mesh convergence analysis performed by Habla et al. [18], and had a similar refinement level to the two most refined meshes (M3 and M4) therein. The expansion or contraction geometrical factors were defined for each direction as the ratio of two consecutive cells lengths ($f_{z}=\Delta z_{i+1}/\Delta z_{i}$, with $\Delta z_{i}$ being the length of the cell *i* in the *z*-direction). In this way, since $f_{z}>1$ in Block V (see Table 1), in the *z* direction, the cells expanded from left to right. Figure 2c shows the details of the level of mesh refinement at the contraction region for M2. The higher refinement that occurs near the walls and in the contraction region allowed for the highest gradients of the computed flow variables in these locations to be captured.

The following boundary and initial conditions were used for all the runs that were performed:

- For velocity, no-slip at the walls, symmetry at the centerline, parabolic velocity profile at the inlet (with average velocity ${\bar{U}}_{z,1}=0.00129$ m/s), and a zero-gradient condition at the outlet, i.e., assuming a fully developed flow;
- For pressure, the inlet and wall boundary conditions were set as zero-gradient and the centerline as symmetry boundary condition. At the outlet Dirichlet boundary condition was used, with a fixed value p=0. Notice that, although the zero-pressure gradient specified at the inlet did not match the fully developed Poiseuille flow with the average velocity ${\bar{U}}_{z,1}$, this inconsistency did not affect the results, because the length of the upstream channel was sufficiently large to achieve fully developed flow conditions;
- For the log-conformation tensor components, zero values were assumed at the inlet, a symmetry boundary condition was used at the centerline, a linear extrapolation of the tensor components to the boundary was used at the walls, and a zero-gradient condition was imposed at the outlet;
- For the temperature, a Dirichlet condition was imposed at the inlet ($T_{inl}=462$ K), a symmetry boundary condition was used at the centerline, at the upstream wall, $\left(z<l_{1}\right)$, $T_{w,1}=462$ K, while, for the downstream wall, $\left(z\geq l_{1}\right)$ the temperature $T_{w,2}$ was chosen such as to give temperature jumps of $\Delta T=T_{w,2}-T_{w,1}=-30\;K,0\;K,30\;K$. A zero-gradient condition was imposed at the outlet;
- All fields were set to zero at the initial time.

### 4.2. Numerical Parameters

The dimensionless numbers governing the flow are the Reynolds number Re, the Weissenberg number Wi, the Peclet number Pe and the retardation ratio β, defined as:

(65) $$\begin{array}{c} \begin{array}{c} Re=\frac{\rho R_{2}{\bar{U}}_{z,2}}{\eta_{0}} \end{array} \end{array}$$

(66) $$\begin{array}{c} \begin{array}{c} Wi=\frac{\lambda {\bar{U}}_{z,2}}{R_{2}} \end{array} \end{array}$$

(67) $$\begin{array}{c} \begin{array}{c} Pe=\frac{\lambda R_{2}{\bar{U}}_{z,2}c_{P}}{k} \end{array} \end{array}$$

where ${\bar{U}}_{z,2}$ is the mean velocity in the axial direction in the downstream channel, and λ is the relaxation time. In this case study, $Re=3.9\times 10^{-5}$, corresponding to creeping flow conditions. The retardation ratio was equal to $\beta =\eta_{S}/\eta_{0}=19/20$, thus assuming the solvent contribution to be negligibly small, which approximately recovered an Upper-Convected-Maxwell model. The Peclet number was kept constant at Pe=345 by setting $c_{P}=1500$ J/kg K and k=0.17 W/mK. The WLF parameters were $C_{1}=4.54$ and $C_{2}=150.36$. The split coefficient varied between pure energy elasticity and entropy elasticity, α=0 or 1, respectively.

The use of a normalized time-step $\Delta t/(R_{2}/{\bar{U}}_{z,2})$ of $10^{-4}$ allowed for converged solutions to be obtained for all the runs performed. The maximum local Courant number corresponding to the normalized time-step $10^{-4}$ obtained for the axisymmetric 4:1 sudden contraction is 0.07.

### 4.3. Effects of the Energy Partitioning Parameter α

**Figure 3:**
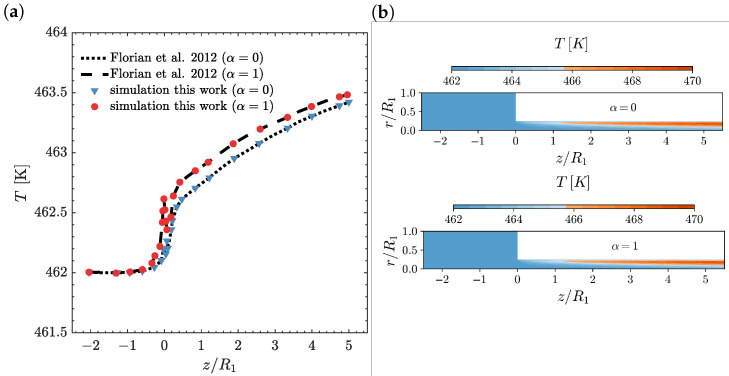
(**a**) Temperature profiles on the line $r=0.97R_{2}$ [18] and (**b**) temperature contours as a function of the axial position $z/R_{1}$ for α=0 and α=1 at Wi=5 and ΔT=0 K using M2.

**Figure 4:**
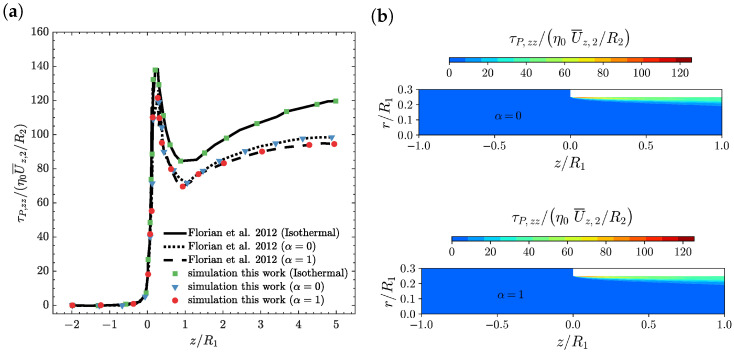
(**a**) Axial normal stress profiles $\tau_{P,zz}/\left(\eta_{0}{\bar{U}}_{z,2}/R_{2}\right)$ on the line $r=0.97R_{2}$ [18] and (**b**) axial normal stress contours as a function of the axial position $z/R_{1}$ for α=0 and α=1 at Wi=5 and ΔT=0 K using M2.

In Figure 3, the temperature profile on the line $r=0.97R_{2}$ (Figure 3a) and the temperature contour plots (Figure 3b) are shown as a function of the axial position $z/R_{1}$ for α=0 and α=1 at Wi=5 and ΔT=0 K. As illustrated in Figure 3a, the temperature profile computed by the newly-developed, fully implicit, block-coupled, non-isothermal, viscoelastic, log-conformation tensor-based algorithm is in agreement with the results presented by Habla et al. [18]. Due to the increase in the deformation rate near the contraction, the dissipation also increases, which leads to a temperature rise shortly before the contraction. At the contraction, the fluid moves to the wall with the imposed fixed wall temperature $T_{w,2}=462$ K and, therefore, due to heat conduction towards the wall, a fast decrease in temperature is observed. Note that this decrease is remarkably higher for entropy elasticity (α=1) due to its higher temperature, which promotes a larger heat conduction rate. Subsequently, just after the re-entrant corner, due to the larger normal stresses developed here (see Figure 4), which lead to an increase of dissipation, the temperature rises again at the steepest rate. Further along the downstream channel, the temperatures also increase, but now at a smaller rate and, ultimately, the difference in the temperatures between energy elasticity and entropy elasticity cases is small, because the energy is now more released as pure energy elasticity (α=0) [18]. The temperature contour plots shown in Figure 3b are similar for both the energy and entropy elasticities, as expected from the marginal differences shown in Figure 3a for the temperature profile at $r=0.97R_{2}$. In both cases, we see the formation of a larger temperature-rising region for $z/R_{1}>1$, which is also extended through the downstream channel radial direction.

In Figure 4, the axial normal stress profile on the line $r=0.97R_{2}$ (Figure 4a) and the axial normal stress contour plots (Figure 4b) as a function of the axial position $z/R_{1}$ for α=0 and α=1 at Wi=5 and ΔT=0 K, are shown. As illustrated in Figure 4a, the axial normal stress profile computed by the newly-developed, fully implicit, block-coupled, non-isothermal, viscoelastic, log-conformation tensor-based algorithm and the isothermal version is in agreement with the results presented by Habla et al. [18]. In the non-isothermal cases, the axial normal stress is smaller than the one obtained for the isothermal calculation. This behaviour can be attributed to the fact that increasing the temperature leads to a reduction in the viscosity value (see Equation (9)), which decreases the stress values, and also leads to a reduction in the relaxation time, resulting in a smaller local Weissenberg number and, therefore, fewer elastic effects (i.e., decrease in stresses). In addition, just after the re-entrant corner, we see an abrupt increase of the normal stresses due to the increase in the fluid deformation in this region, followed by a fast relaxation, before it starts to increase further down the downstream channel, but now at a smaller rate. The axial normal stress contour plots shown in Figure 4b are again similar for both the energy and entropy elasticities. In both cases, we see the formation of a thinner layer of normal stresses rising region at $0<z/R_{1}<0.2$, which then increases in width, but with smaller normal stress values, at $0.2<z/R_{1}<1$.

**Figure 5:**
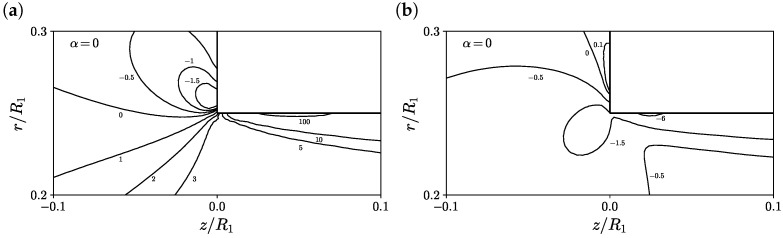
Contours of (**a**) first normal stress difference $\left(\tau_{P,zz}-\tau_{P,rr}\right)/\left(\eta_{0}{\bar{U}}_{z,2}/R_{2}\right)$ and (**b**) shear stress $\tau_{P,rz}/\left(\eta_{0}{\bar{U}}_{z,2}/R_{2}\right)$ for α=0, Wi=5 and ΔT=0 K using M2.

Additionally, Figure 5 shows the contours of the first normal stress difference and shear stress predicted on M2 at α=0, Wi=5 and ΔT=0 K, for a zoomed region around the re-entrant corner. The maximum first normal stress difference is located on the downstream channel wall near the re-entrant corner (see Figure 5a). Moreover, at the contraction vertical wall, the first normal stress difference is negative, which is responsible for fluid re-circulation and the formation of the corner vortex. The maximum magnitude of the shear stress component is also located on the downstream channel wall near the re-entrant corner (see Figure 5b); however, in that case, the shear stress value is negative, pushing the fluid towards the symmetry line at $r/R_{1}=0$. Finally, at the contraction vertical wall, the shear stress is positive, allowing for the extension of the corner vortex size.

**Figure 6:**
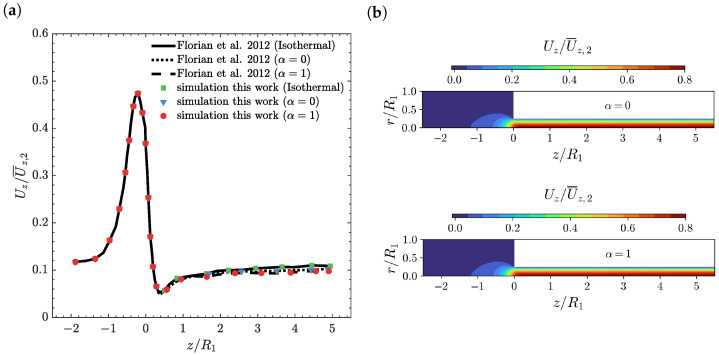
(**a**) Axial velocity profiles $U_{z}/{\bar{U}}_{z,2}$ on the line $r=0.97R_{2}$ [18] and (**b**) axial velocity contours as a function of the axial position $z/R_{1}$ for α=0 and α=1 at Wi=5 and ΔT=0 K using M2.

In Figure 6, the axial velocity profile on the line $r=0.97R_{2}$ (Figure 6a) and the axial velocity contour plots (Figure 6b) as a function of the axial position $z/R_{1}$ for α=0 and α=1 at Wi=5 and ΔT=0 K are shown. As illustrated in Figure 6a, the axial velocity profiles computed by the newly-developed, fully implicit, block-coupled, non-isothermal, viscoelastic, log-conformation tensor-based algorithm and with the isothermal version are in agreement with the results presented by Habla et al. [18]. In addition, we can see that the influence of temperature on the local velocity profile for both cases of entropy elasticity and energy elasticity is negligible, being similar to the axial velocity of the isothermal case. The axial velocity contour plots shown in Figure 6b are, again, similar for both the energy and entropy elasticities, showing the accommodation of the fluid near the re-entrant corner, i.e., the fluid is accelerated in the center while decelerating in the wall-near regions, and a fully developed velocity profile at the downstream channel.

In Table 2, we provide a summary of the number of iterations and execution time of the segregated/iterative and coupled/monolithic approaches for the calculation of the non-isothermal, viscoelastic matrix-based Oldroyd-B fluid flow in the two-dimensional, axisymmetric 4:1 planar sudden-contraction geometry, using the two different meshes, M1 and M2, with α=0 at Wi=5 and ΔT=0 K. The ratio of the number of iterations required by the segregated algorithm to that required by the coupled one increases from 432 to 524 for M1 and M2, respectively, with accompanying computational cost ratios of 17 and 19, respectively, which clearly shows the benefits obtained by using the fully implicit coupled approach.

### 4.4. Effects of Wall Temperature Jumps

**Figure 7:**
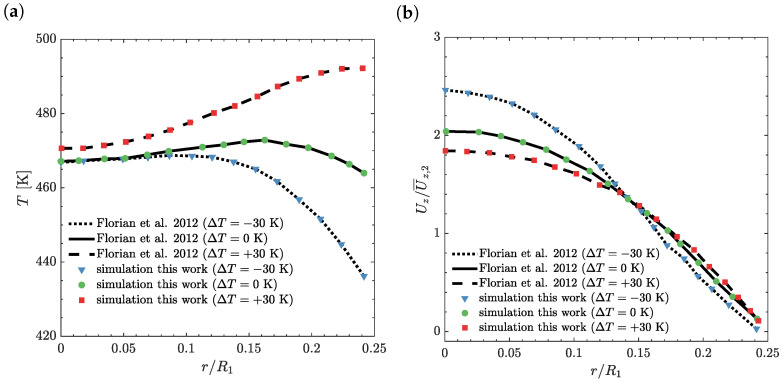
(**a**) Temperature *T* and (**b**) axial velocity $U_{z}/{\bar{U}}_{z,2}$ as a function of the radial distance $r/R_{1}$ at the outlet, with Wi=5, α=0 and temperature jumps ΔT=−30 K, ΔT=0 K and ΔT=+30 K using M2 [18].

The temperature and axial velocity profiles at the outlet of the downstream section are shown in Figure 7 for Wi=5, α=0 and temperature jumps ΔT=−30 K, ΔT=0 K and ΔT=+30 K. The results for the case of energy elasticity at the outlet of the downstream section, for all temperature jumps, did not present any differences [18]. As shown in Figure 7a, the wall ($r/R_{1}=0.25$) temperature changes by more than 60 K and the centerline ($r/R_{1}=0$) temperature varied less than 5 K from ΔT=−30 K to ΔT=+30 K, in agreement with the results obtained by Habla et al. [18]. These temperature changes are responsible for the limited effect of external heating or cooling in the thermal control of the flow at the bulk region. In Figure 7b, the axial velocity profile at the centerline increases with the decrease in temperature jump, due to the smaller viscosity in the near-wall region.

**Figure 8:**
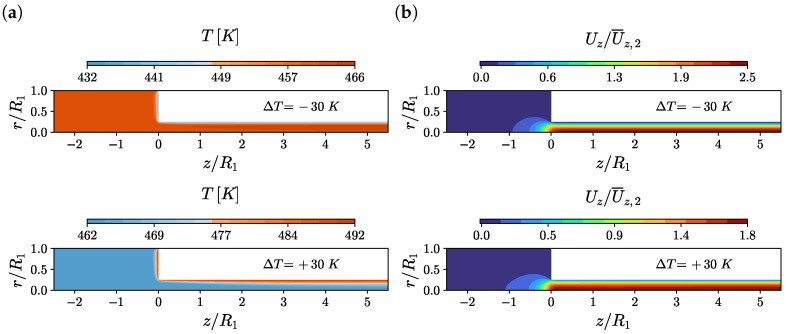
(**a**) Temperature *T* and (**b**) axial velocity $U_{z}/{\bar{U}}_{z,2}$ contours at Wi=5, α=0 and temperature jumps ΔT=−30 K and ΔT=+30 K using M2.

The temperature and axial velocity contours are shown in Figure 8 for Wi=5, α=0 and temperature jumps ΔT=−30 K and ΔT=+30 K. For the cooling case, i.e., ΔT=−30 K (top figures in Figure 8), the reduction in temperature on the downstream channel walls promotes an increase in fluid centerline velocity of approximately 3.1 times more than the one obtained for the case ΔT=0 K (see Figure 6). For the heating case, i.e., ΔT=+30 K (bottom figures in Figure 8), the increase in temperature on the downstream channel walls promotes an increase in fluid centerline velocity of approximately 2.3 times more than the one obtained for the case ΔT=0 K (see Figure 6). Notice that the increase in centerline velocity for the heating case is approximately 75% smaller than the one obtained for the cooling case.

**Figure 9:**
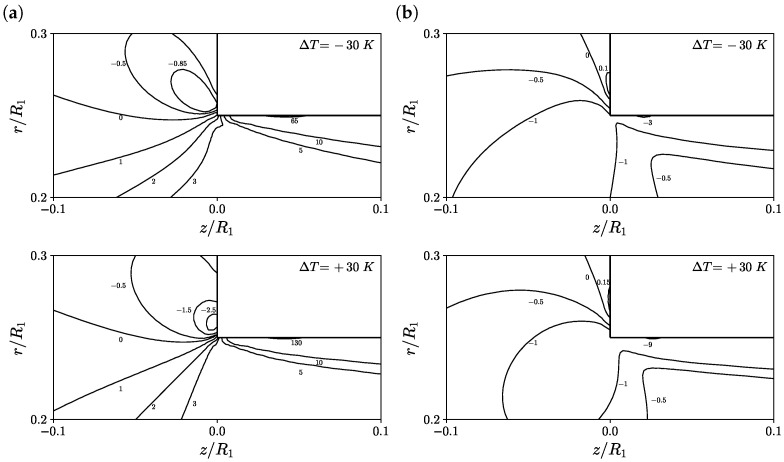
Contours of (**a**) first normal stress difference $\left(\tau_{P,zz}-\tau_{P,rr}\right)/\left(\eta_{0}{\bar{U}}_{z,2}/R_{2}\right)$ and (**b**) shear stress $\tau_{P,rz}/\left(\eta_{0}{\bar{U}}_{z,2}/R_{2}\right)$ for α=0, Wi=5, ΔT=−30 K (top) and ΔT=+30 K (bottom) using M2.

Additionally, Figure 9 shows the contours of the first normal stress difference and shear stress predicted on M2 at α=0, Wi=5, ΔT=−30 K (top) and ΔT=+30 K (bottom), for a zoomed region around the re-entrant corner. The maximum first normal stress difference was found to decrease by 35% and increase by 30% for the cases ΔT=−30 K and ΔT=+30 K, respectively, when compared to the case ΔT=0 K. The minimum value of the shear stress is found to both increase and decrease by 50% for the cases ΔT=−30 K and ΔT=+30 K, respectively, when compared to the case ΔT=0 K.

## 5. Conclusions

This paper presented a fully implicit coupled method (also known as a monolithic approach) for the solution of laminar, incompressible, non-isothermal, viscoelastic flows based on the log-conformation tensor framework for high Weissenberg number problems. The fully implicit coupled solver is a pressure-based method in which the pressure equation is derived through the Rhie–Chow interpolation, allowing for coupling between the pressure and velocity fields. In addition, the explicit diffusion term added by the improved both-sides-diffusion (iBSD) technique to the linear momentum equations is discretized with a special second-order derivative of the velocity field, allowing for coupling between the velocity and log-conformation tensor fields. Subsequently, the divergence of the log-conformation tensor term in the linear momentum equations is implicitly discretized, and the velocity field is considered implicitly in the log-conformation tensor constitutive equations by expanding the advection, rotation and the rate of deformation terms, all by considering a Taylor series expansion truncated at the second-order error term. Finally, the advection and diffusion terms in the energy equation are also implicitly discretized.

The validation of the newly-developed algorithm was performed for the non-isothermal viscoelastic matrix-based Oldroyd-B fluid flow in the benchmark problem of a two-dimensional axisymmetric 4:1 planar sudden-contraction geometry, and the results obtained by the fully implicit coupled algorithm were shown to be in remarkable agreement with other results found in the scientific literature (less than 5% error differences), which validated the present implementation. The results were obtained at a high Weissenberg number, and allowed to study the influence of the energy splitting factor at the limit of pure energy elasticity and pure entropy elasticity, and the effect of the wall temperature jump near the contraction for positive and negative temperature increments.

In future works, the algorithm will be further assessed by looking at 3D case studies and employing non-linear viscoelastic fluid behaviors, such as the shear-thinning phenomenon predicted by the Giesekus fluid model. 

## Figures and Tables

**Table 1:** Characteristics of the meshes employed to simulate the non-isothermal viscoelastic flow of a viscoelastic matrix fluid described by the Oldroyd-B constitutive model in the axisymmetric 4:1 planar sudden contraction geometry.

Block	Mesh 1 (M1)			Mesh 2 (M2)		
	Nz×Nr	$f_{z}$	$f_{r}$	Nz×Nr	$f_{z}$	$f_{r}$
Block I	61×20	0.9061	0.9206	122×40	0.9519	0.9595
Block II	61×25	0.9061	1.0996	122×50	0.9519	1.0486
Block III	61×8	0.9061	0.8593	122×16	0.9519	0.9270
Block IV	40×20	1.1036	0.9206	80×40	1.0505	0.9595
Block V	13×20	1.1740	0.9206	26×40	1.0835	0.9595
NC	4293				17172	
$\Delta z_{\min}=\Delta r_{\min}$	$0.02R_{2}$			$\Delta z_{\min}=\Delta r_{\min}$	$0.01R_{2}$	

Nz and Nr are number of cells along *z* and *r* directions, respectively, inside each block. *f_z_* and *f_r_* are the expansion/contraction ratios inside each block. NC is the number of cells in the mesh. Δ*z*_min_ and Δ*r*_min_ are the minimum cell size in each direction.

**Table 2:** Comparison of the number of iterations and CPU time required by the segregated (S) and coupled (C) solvers for the calculation of the non-isothermal viscoelastic matrix-based Oldroyd-B fluid flow in the two-dimensional axisymmetric 4:1 planar sudden-contraction geometry, using the two different meshes M1 and M2, with α=0 at Wi=5 and ΔT=0 K.

Mesh	Number of Iterations			Execution Time [s]		
	C	S	S/C	C	S	S/C
M1	827	357,681	432	103	1760	17
M2	1484	778,344	524	689	13,102	19

M1: 4293 CV; M2: 17172 CV.

## Data Availability

Not applicable.
